# The SH3BGR/STAT3 Pathway Regulates Cell Migration and Angiogenesis Induced by a Gammaherpesvirus MicroRNA

**DOI:** 10.1371/journal.ppat.1005605

**Published:** 2016-04-29

**Authors:** Wan Li, Qin Yan, Xiangya Ding, Chenyou Shen, Minmin Hu, Ying Zhu, Di Qin, Hongmei Lu, Brian J. Krueger, Rolf Renne, Shou-Jiang Gao, Chun Lu

**Affiliations:** 1 State Key Laboratory of Reproductive Medicine, Nanjing Medical University, Nanjing, People’s Republic of China; 2 Key Laboratory of Pathogen Biology of Jiangsu Province, Nanjing Medical University, Nanjing, People’s Republic of China; 3 Department of Microbiology, Nanjing Medical University, Nanjing, People’s Republic of China; 4 Department of Molecular Microbiology and Immunology, Keck School of Medicine, University of Southern California, Los Angeles, California, United States of America; 5 Department of Obstetrics, The First Affiliated Hospital of Nanjing Medical University, Nanjing, People’s Republic of China; 6 Department of Molecular Genetics and Microbiology, University of Florida, Gainesville, Florida, United States of America; Wistar Institute, UNITED STATES

## Abstract

Kaposi’s sarcoma (KS)-associated herpesvirus (KSHV) is a gammaherpesvirus etiologically associated with KS, a highly disseminated angiogenic tumor of hyperproliferative spindle endothelial cells. KSHV encodes 25 mature microRNAs but their roles in KSHV-induced tumor dissemination and angiogenesis remain unknown. Here, we investigated KSHV-encoded miR-K12-6-3p (miR-K6-3p) promotion of endothelial cell migration and angiogenesis, which are the underlying mechanisms of tumor dissemination and angiogenesis. We found that ectopic expression of miR-K6-3p promoted endothelial cell migration and angiogenesis. Mass spectrometry, bioinformatics and luciferase reporter analyses revealed that miR-K6-3p directly targeted sequence in the 3’ untranslated region (UTR) of SH3 domain binding glutamate-rich protein (SH3BGR). Overexpression of SH3BGR reversed miR-K6-3p induction of cell migration and angiogenesis. Mechanistically, miR-K6-3p downregulated SH3BGR, hence relieved STAT3 from SH3BGR direct binding and inhibition, which was required for miR-K6-3p maximum activation of STAT3 and induction of cell migration and angiogenesis. Finally, deletion of miR-K6 from the KSHV genome abrogated its effect on the SH3BGR/STAT3 pathway, and KSHV-induced migration and angiogenesis. Our results illustrated that, by inhibiting SH3BGR, miR-K6-3p enhances cell migration and angiogenesis by activating the STAT3 pathway, and thus contributes to the dissemination and angiogenesis of KSHV-induced malignancies.

## Author Summary

Kaposi’s Sarcoma (KS), caused by infection of Kaposi’s sarcoma (KS)-associated herpesvirus (KSHV), is a tumor of endothelial cells characterized by angiogenesis and invasiveness. *In vitro*, KSHV-infected endothelial cells display an increased invasiveness and angiogenicity. KSHV encodes twelve precursor miRNAs (pre-miRNAs), which are processed into at least 25 mature miRNAs. However, the roles of these miRNAs in KSHV-induced tumor dissemination and angiogenesis remain unknown. Here, we investigated KSHV-encoded miR-K12-6-3p (miR-K6-3p) promotion of endothelial cell migration and angiogenesis, which are the underlying mechanisms of tumor dissemination and angiogenesis. We demonstrated that miR-K6-3p promoted cell migration and angiogenesis by directly targeting SH3 domain binding glutamate-rich protein (SH3BGR). Furthermore, we found that STAT3, which was negatively regulated by SH3BGR mediated miR-K6-3p-induced cell migration and angiogenesis. MiR-K6-3p downregulation of SH3BGR, hence relieved SH3BGR direct inhibition of STAT3 resulting in the activation of STAT3 and induction of cell migration and angiogenesis. These results identify miR-K6-3p and its the downstream pathway as potential therapeutic targets for the treatment of KSHV-associated malignancies.

## Introduction

Kaposi’s sarcoma-associated herpesvirus (KSHV) is a gammaherpesvirus associated with AIDS-associated Kaposi’s sarcoma (KS), primary effusion lymphoma (PEL), and multicentric Castleman’s disease (MCD) [1]. KS is an angiogenic vascular tumor of endothelial spindle cells [2]. KS is characterized by vast aberrant proliferation of small vessels, lack of basement membrane, and excessive leakiness with microhemorrhages and hemosiderin deposition [3]. Although skin lesions are a common manifestation of KS, KS is also a highly disseminated tumor often observed as multifocal lesions and in visceral organs. KS spindle cells have increased invasiveness, which has been attributed to the enhanced expression of several matrix metalloproteinases (MMPs), including MMP-1, MMP-2, MMP-3, MMP-7, MMP-9, and MMP-13 [4–7]. In KS lesions, the majority of the spindle tumor cells are latently infected by KSHV; however, a small number of them also undergo spontaneous lytic replication. Lytic replication generates infectious virions for spreading to other cells and at the same time produces virus-encoded cytokines as well as induces cellular cytokines through viral lytic proteins or de novo viral infection, all of which could contribute to KS pathogenesis by promoting KS angiogenesis, inflammatory infiltration and tumor dissemination through autocrine and paracrine mechanisms [8].

MicroRNAs (miRNAs) are a class of ~22 nt long non-coding small RNAs involved in diverse cellular functions and in all phases of cancer development [9]. They act post-transcriptionally to regulate the expression of large numbers of genes by targeting the complementary gene sequences through its seed region. Usually, miRNAs bind to complementary sequences within the 3’ untranslated region (UTR) of a target gene resulting in mRNA degradation or down-regulation of translation [10]. KSHV encodes 25 mature microRNAs derived from 12 precursor miRNAs (pre-miRs), which are highly expressed during viral latency and in KS tumors [1, 11–16]. KSHV miRNAs not only promote viral latency by directly targeting viral genes or indirectly targeting cellular pathways [17–24], but also modulate apoptosis, cell cycle, cytokine production and secretion, immune evasion, epigenetics, cellular transformation, and angiogenesis by directly regulating KSHV and/or host genes [18, 25–43]. For example, KSHV miR-K6-5p represses breakpoint cluster region protein expression, enhances Rac1 activity, and increases *in vitro* angiogenesis [40]. Furthermore, miR-K2 and -K5 inhibit tropomyosin 1 and increase anchorage-independent growth and endothelial tube formation [42]. Besides angiogenesis, KSHV miRNAs are also involved in cell motility. Our recent study has shown that, by directly targeting G protein-coupled receptor (GPCR) kinase 2 (GRK2), miR-K3 promotes endothelial cell migration and invasion via activation of the CXCR2/AKT signaling pathway, which might contribute to the dissemination of KSHV-induced tumors [44].

SH3 domains are protein–protein interaction modules that recognize poly-proline motifs in a context dependent manner [45]. These SH3 domains containing adaptors have been implicated in diverse processes including mediation of signaling induced by growth factors, cytoskeletal regulation, vesicle trafficking, membrane dynamics, cell motility, endocytosis, and cell adhesion [45–47]. These processes are crucial in regulating different aspects of cancer cell homeostasis [47]. SH3 domain binding glutamate-rich protein (SH3BGR), which contains a highly conserved SH3 binding motif and a glutamic acid-rich domain at the COOH terminal [48], was initially identified to be involved in heart morphogenesis, and hence, in the pathogenesis of congenital heart disease (CHD) in Down syndrome (DS) [49]. Furthermore, SH3BGR was also implicated in obesity [50]. However, the role of SH3BGR in the pathogenesis of cancer remains unclear.

Because miR-K6-3p is expressed at high level in B cells latently infected by KSHV [51] and in KS tumors [52], we set out to examine the effect of miR-K6-3p on cell mobility and angiogenesis. We found that miR-K6-3p directly targeted SH3BGR to promote endothelial cell migration and angiogenesis. Furthermore, activation of the STAT3 pathway, which was negatively regulated by SH3BGR, contributed to miR-K6-3p-induced endothelial cell migration and angiogenesis. To our knowledge, this is the first report to describe the involvement of a viral miRNA in both cell migration and angiogenesis. Because of the high angiogenicity and invasiveness of KS, our findings reveal a novel mechanism by which KSHV miRNAs contribute to the pathogenesis of KSHV-associated tumors.

## Results

### Ectopic Expression of miR-K6-3p Promotes Endothelial Cell Migration and Angiogenesis

**Fig 1:**
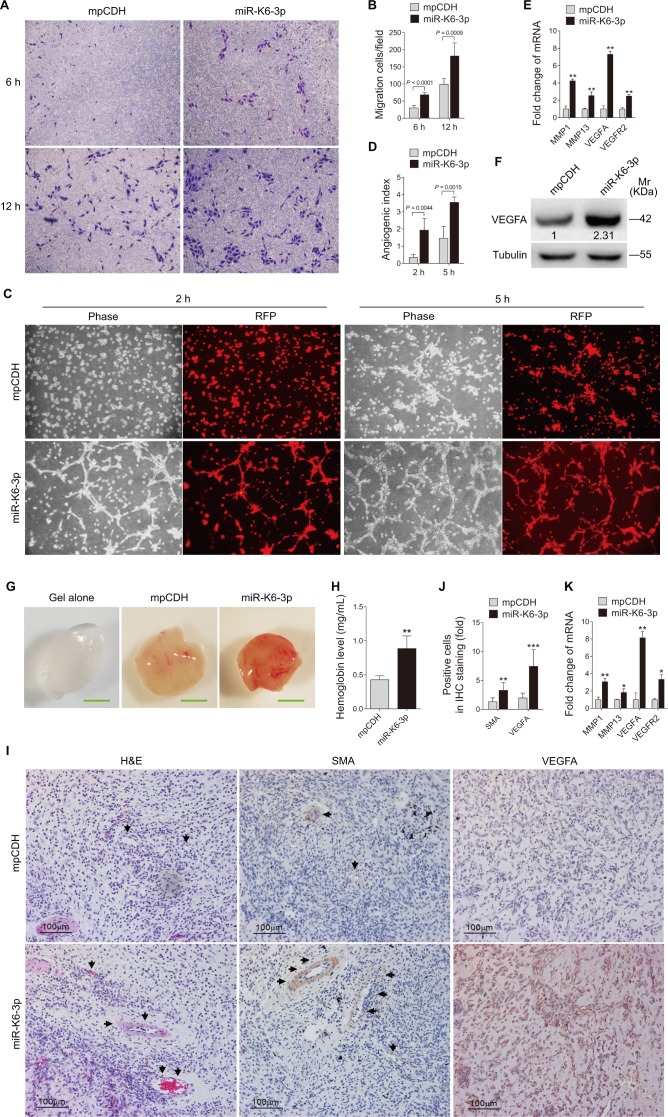
Ectopic expression of miR-K6-3p promotes endothelial cell migration and angiogenesis. **(A)**. Transwell migration for HUVEC transduced with 1 MOI lentivirus empty vector (**mpCDH**) or lentivirus-miR-K6-3p (**miR-K6-3p**). The representative images were captured at 6 and 12 h post seeding (original magnification, ×100). **(B)**. The quantification results of Transwell migration assay in (**A**). The quantified results represent the mean ± SD. Three independent experiments were performed and similar results were obtained, each experiment containing at least four technical replicates. **(C)**. Microtubule formation assay for HUVEC transduced with lentivirus empty vector (**mpCDH**; top) or lentivirus-miR-K6-3p (**miR-K6-3p**; bottom). The representative images were captured under the light microscope (**Phase**) and fluorescent microscope (**RFP**) at 2 and 5 h post seeding (original magnification, ×100). **(D)**. The quantification results of microtubule formation assay in (**C**). The quantified results represent the mean ± SD. Three independent experiments were performed and similar results were obtained, each experiment containing four technical replicates. **(E)**. The mRNA expression of MMP1, MMP13, VEGFA and VEGFR2 in HUVEC treated as in (**A**) was determined by RT-qPCR. The quantified results represent the mean ± SD. Three independent experiments were performed and similar results were obtained, each experiment containing four technical replicates. ** *P* < 0.01 for Student’s *t*-test versus mpCDH group. **(F)**. Western blotting analysis of the expression of VEGFA protein in HUVEC treated as in (**A**). Results shown were from a representative experiment of three independent experiments with similar results. The values of density of protein bands after normalization to housekeeping were shown. **(G)**. HUVEC treated as in (**A**) were examined for their proangiogenic effects in Matrigel plug assay in nude mice as described in the “Materials and Methods” section. Representative photographs of angiogenesis in the nude mice are shown. **(H)**. The hemoglobin level of the Matrigel plugs treated as in (**G**) was determined with hemoglobin content calculated based on the standard curve. Data represent mean ± SD, each group with five tumors (n = 5). Three independent experiments were performed and similar results were obtained. ** *P* < 0.01 for Student’s *t*-test versus mpCDH group. **(I)**. Hematoxylin and eosin staining analysis of histologic features (**left**; ×400) and immunohistochemical (IHC) staining analysis of the expression of SMA (**middle**; ×400) and VEGFA (**right**; ×400) in plugs induced by HUVEC transduced with mpCDH or miR-K6-3p. Black arrows point to neovascularization and hemorrihagic foci in H&E staining sections and the SMA in IHC staining sections, respectively. **(J)**. Quantification of results in (**I**). ** *P* < 0.01 and *** *P* < 0.001 for Student’s *t*-test versus mpCDH group. **(K)**. The mRNA expression of MMP1, MMP13, VEGFA and VEGFR2 in the Matrigel plugs treated as in **(G)** were determined by RT-qPCR. The quantified results represent the mean ± SD. Three independent experiments were performed and similar results were obtained, each experiment containing four technical replicates. ** *P* < 0.01 and *** *P* < 0.001 for Student’s *t*-test versus mpCDH group.

To examine the involvement of miR-K6-3p in endothelial cell motility and angiogenesis, we transduced HUVEC with the different MOIs of a lentivirus expressing miR-K6-3p. At MOI 1, miR-K6-3p-transduced HUVEC showed a miR-K6-3p expression level similar to that of KSHV (BAC16)-infected HUVEC (S1A Fig). Thus, we chose MOI 1 for the subsequent transduction experiments. Under this condition, over 94% cells were RFP-positive at day 3 or 4 post-transduction, indicating the successful lentivirus transduction (S1B and S1C Fig). Expectedly, miR-K6-3p markedly inhibited the activity of pGL3-miR-K6-3p sensor reporter, indicating that the miR-K6-3p expression construct was functional in HUVEC (S1D Fig). To examine the effect of miR-K6-3p on cell motility and invasion, Transwell migration and Matrigel invasion assays were performed with miR-K6-3p-expressing HUVEC. As shown in Fig 1A and 1B, HUVEC transduced with miR-K6-3p exhibited strikingly enhanced abilities of migration when compared with cells transduced with the vector control. However, miR-K6-3p did not induce cell invasion. To determine the effect of miR-K6-3p on angiogenesis, we first performed a microtubule formation assay. We found that ectopic expression of miR-K6-3p dramatically increased tube formation in HUVEC compared to control vector (Fig 1C and 1D). Furthermore, RT-qPCR was performed to detect several cytokines that are related to cell migration and angiogenesis. We found that ectopic expression of miR-K6-3p in HUVEC increased the expression levels of MMP1 and MMP13 mRNA transcripts as well as those of VEGFA and VEGFR2 (Fig 1E). Western blotting showed that miR-K6-3p strongly promoted the expression of VEGFA (Fig 1F).

To further confirm the effect of miR-K6-3p on angiogenesis, Matrigel plug assay was performed. By detecting the hemoglobin content in the plug, which represented the relative angiogenesis index, we found that miR-K6-3p significantly increased the angiogenesis in nude mice (Fig 1G and 1H). Hematoxylin and eosin (H&E) staining showed extensive dense neovascularization and hemorrhagic necrotic foci in the miR-K6-3p plugs. There were more smooth muscle actin (SMA)- and VEGFA-positive cells in plugs induced by miR-K6-3p (Fig 1I and 1J). qPCR was used to measure the transcriptional levels of cytokines in plugs. As expected, the levels of MMP1, MMP13, VEGFA, and VEGFR2 mRNAs were significantly elevated in plugs of miR-K6-3p-transduced HUVEC (Fig 1K). Taken together, these data suggest that miR-K6-3p promotes endothelial cell migration and angiogenesis.

### miR-K6-3p Induces Migration and Angiogenesis of Endothelial Cells by Directly Targeting SH3BGR

**Fig 2:**
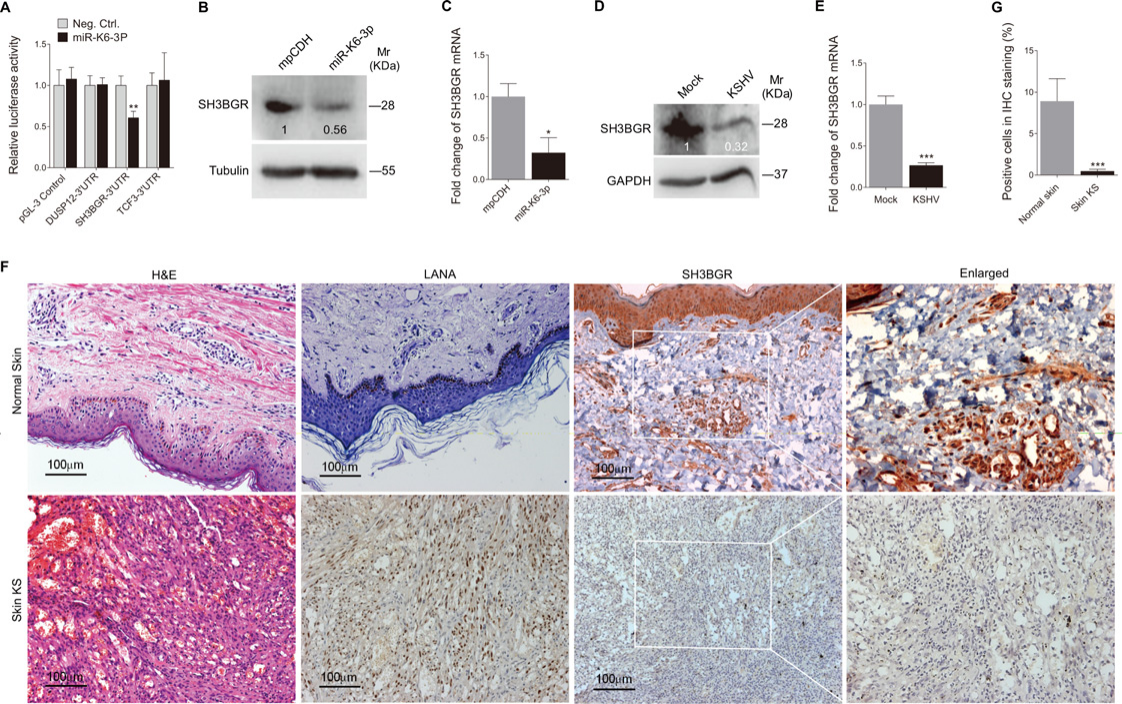
SH3BGR expression is reduced in miR-K6-3p-expressing HUVEC and KS lesion samples. **(A)**. Luciferase activity was detected in HEK293T cells co-transfected by a mimic of miR-K6-3p (**miR-K6-3p**) or a negative control nucleotide of miRNA (**Neg. Ctrl.**) together with pGL3-Control (**pGL3-Control**), pGL3-DUSP12 3'UTR luciferase reporter (**DUSP12-3’UTR**), pGL3-SH3BGR 3’UTR luciferase reporter (**SH3BGR-3’UTR**) or pGL3-TCF3 3’UTR luciferase reporter (**TCF3-3’UTR**) for 24 h. The relative reporter activity level of pGL3-Control, DUSP12-3'UTR, SH3BGR-3’UTR or TCF3-3’UTR in the Neg. Ctrl. group was set as ‘‘1” for comparison, respectively. ** *P* < 0.01 for Student’s *t*-test versus Neg. Ctrl. group. **(B)**. The expression of SH3BGR protein in HUVEC transduced with lentivirus empty vector (**mpCDH**) or lentivirus-miR-K6-3p (**miR-K6-3p**) was detected by Western blotting. Results shown were from a representative experiment of three independent experiments with similar results. The values of density of protein bands after normalization to housekeeping were shown. **(C)**. The mRNA level of SH3BGR in HUVEC transduced with lentivirus empty vector (**mpCDH**) or lentivirus-miR-K6-3p (**miR-K6-3p**) was examined by qPCR. The quantified results represent the mean ± SD. Three independent experiments were performed and similar results were obtained, each experiment containing four technical replicates. * *P* < 0.05 for Student’s *t*-test versus mpCDH group. **(D)**. Western blotting analysis of the expression of SH3BGR protein in KSHV-infected HUVEC (**KSHV**) as described in the 'Materials and Methods' section or in HUVEC treated with PBS as the negative control (**Mock**). Results shown were from a representative experiment of three independent experiments with similar results. The values of density of protein bands after normalization to housekeeping were shown. **(E)**. qPCR analysis for SH3BGR mRNA in KSHV-infected HUVEC (**KSHV**) as described in the 'Materials and Methods' section or in HUVEC treated with PBS as the negative control (**Mock**). The quantified results represent the mean ± SD. Three independent experiments were performed and similar results were obtained, each experiment containing four technical replicates. *** *P* < 0.001 for Student’s *t*-test versus Mock group. **(F)**. Hematoxylin and eosin (H&E) staining of KS lesion (**bottom**) and normal skin (**top**) to show histologic features (left panel; original magnification, ×200) and immunohistochemical (IHC) staining of KSHV LANA and SH3BGR (middle and right panels, respectively; original magnification, ×200). **(G)**. Quantification of results in **(F)**. *** *P* < 0.001 for Student’s *t*-test versus Normal skin group.

To identify the targets of miR-K6-3p, we examined proteins differential expressed between miR-K6-3p- and mpCDH-transduced HUVEC by mass spectrometry analysis. Among the altered cellular proteins, 47 were downregulated by > 2.0 folds by miR-K6-3p (Table 1). Bioinformatics analysis with several programs including TargetScan, RNAhybrid, Findtar, and Pita, was then performed to predict the putative miR-K6-3p targets in these 47 proteins. As shown in Table 2, we predicted 3 proteins that might have miR-K6-3p putative binding sites in their 3'UTR. A luciferase reporter assay confirmed that miR-K6-3p only decreased luciferase activity of the SH3BGR 3’UTR reporter (Fig 2A). Both the protein and mRNA levels of SH3BGR were markedly down-regulated in miR-K6-3p-expressing HUVEC compared to cells expressing the control vector (Fig 2B and 2C). In KSHV-infected HUVEC, both protein and mRNA levels of SH3BGR were also dramatically reduced compared to the mock-infected control (Fig 2D and 2E). Consistent with these observations, there were less SH3BGR-postive cells in KS lesion compared to the normal skin tissue as shown by IHC staining (Fig 2F and 2G).

**Table 1:** Cellular proteins downregulated >2.0 folds in HUVEC infected with miR-K6-3p.

Protein name	Fold	Protein name	Fold	Protein name	Fold	Protein name	Fold
TYMP	-7.87±2.19	CFDP1	-2.61±0.15	TGM2	-2.27±0.02	GINS3	-2.08±0.20
HSPBP1	-4.57±2.43	TPM4	-2.57±0.15	RRM1	-2.25±0.15	PRPS1	-2.06±0.02
CSTF2T	-3.94±1.91	PRPSAP2	-2.51±0.44	TUBB3	-2.24±0.34	PTMS	-2.06±0.06
TCF3	-3.74±0.71	DUSP12	-2.50±1.30	NACA	-2.22±0.74	NRBP1	-2.05±0.03
PLXNB2	-3.44±1.60	PPP1R13L	-2.48±0.60	CDK5	-2.22±0.05	AKR1A1	-2.04±0.12
WRNIP1	-3.38±0.06	MYL6	-2.40±0.10	UFC1	-2.19±0.35	CACYBP	-2.03±0.07
NRD1	-3.17±1.29	PSME2	-2.38±0.04	CFL1	-2.18±0.03	DAGLB	-2.03±0.43
C3orf37	-3.14±0.69	NT5C3L	-2.37±0.48	CAPNS1	-2.15±0.09	TALDO1	-2.03±0.02
NUDT3	-3.10±0.60	PFAS	-2.35±0.12	SUB1	-2.11±0.02	NME1-NME2	-2.02±0.09
PGAM1	-2.93±0.23	RPS12	-2.29±0.03	GSTP1	-2.10±0.41	MAT2B	-2.02±0.11
SH3BGR	-2.89±0.17	TMSB4X	-2.29±0.50	AKT2	-2.10±0.30	EEF2	-2.02±0.01
ARPC3	-2.64±0.70	EDF1	-2.29±0.10	CNN2	-2.09±0.12		

**Table 2:** Fold changes for selected proteins identified in miR-K6-3p-transduced HUVEC using TMT quantification.

Protein name	Fold change			Function	Target prediction		
	Rep1	Rep2	Rep3		Loop score	△G	Recommendation
DUSP12	0.56	0.58	0.25	Cell cycle	12.5	-21.10	OK
SH3BGR	0.37	0.33	0.34	Obesity	25	-19.9	excellent
TCF3	0.29	0.22	0.31	Stem cell self renewal	12.5	-22.5	OK

**Fig 3:**
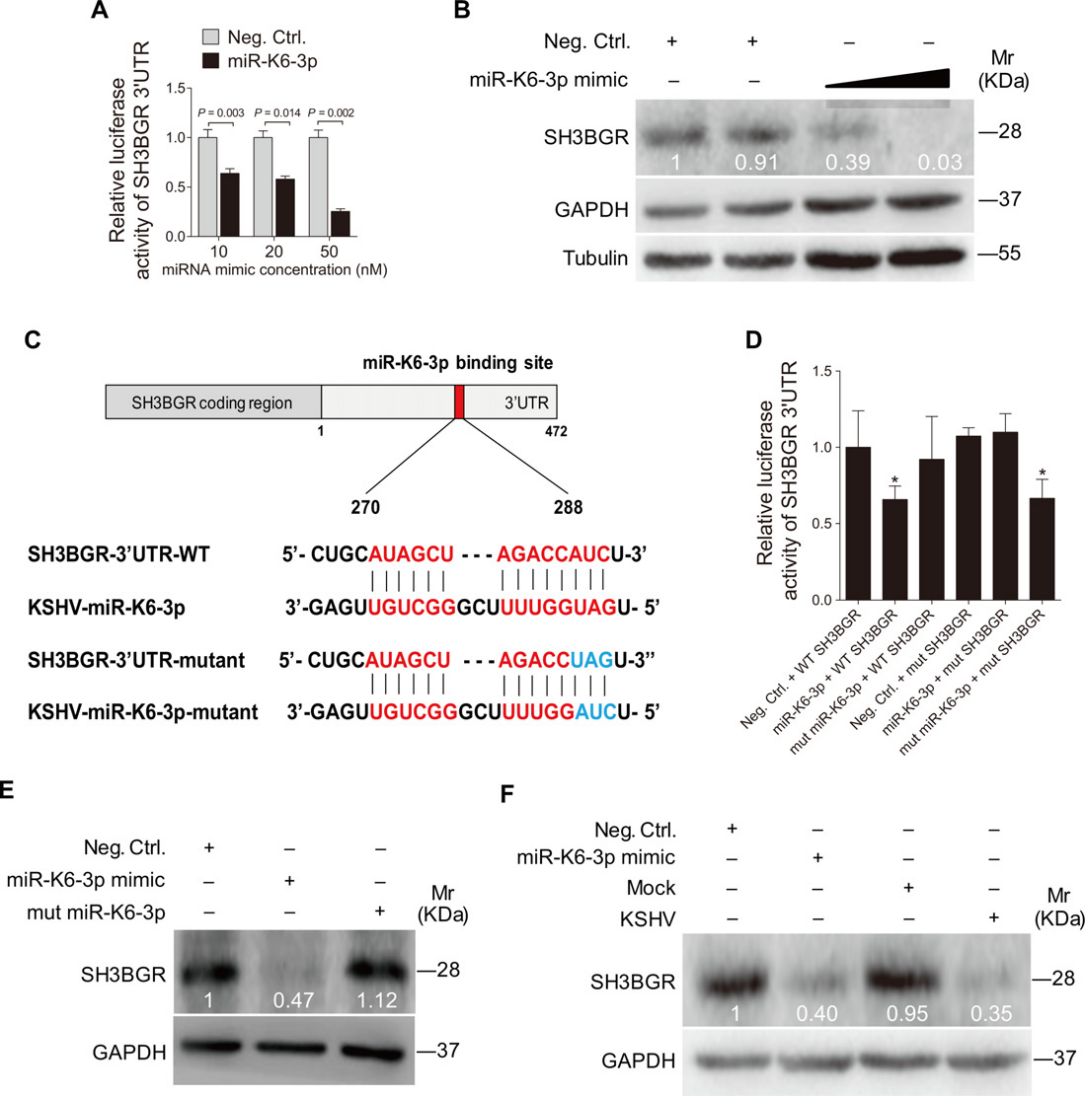
SH3BGR is directly targeted by miR-K6-3p. **(A).** Luciferase assay of HEK293T cells co-transfected with pGL3-SH3BGR 3′UTR reporter together with an increasing amount (10, 20, and 50 nM) of negative control nucleotide of miRNA (**Neg. Ctrl.**) or a mimic of miR-K6-3p (**miR-K6-3p**) for 24 h. **(B).** HUVEC transfected with an increasing amount (20 and 50 nM) mimic of miR-K6-3p or negative control for 48 h. The transfected cells were collected and Western blotting was performed with the indicated antibodies. Results shown were from a representative experiment of three independent experiments with similar results. The values of density of protein bands after normalization to housekeeping were shown. **(C).** Schematic illustration of the putative seed sequences of miR-K6-3p complementary with SH3BGR 3’UTR and mutagenesis of binding sites in the 3’UTR of SH3BGR. **(D).** Effect of mutation of the putative binding site on the SH3BGR 3’UTR reporter. After co-transfection of SH3BGR wild type 3’UTR (**WT SH3BGR**) or the mutant SH3BGR 3’UTR construct (**mut SH3BGR**) together with a negative control nucleotide of miRNA (**Neg. Ctrl.**), a mimic of miR-K6-3p (**miR-K6-3p**) or a mutant mimic of miR-K6-3p (**mut miR-K6-3p**) for 24 h in HEK293T cells, cells were collected and assayed for luciferase activity. * *P* < 0.05 for Student’s *t*-test versus Neg. Ctrl. group. **(E).** Mutant miR-K6-3p failed to target endogenous SH3BGR in HUVEC. A miRNA negative control nucleotide (**Neg. Ctrl.**), a mimic of miR-K6-3p (**miR-K6-3p mimic**; 10 nM) or a mutant mimic of miR-K6-3p (**mut miR-K6-3p**) lacking the seed sequences were transfected into HUVEC for 48 h, respectively. The transfected cells were collected and Western blotting was performed with the indicated antibodies. Results shown were from a representative experiment of three independent experiments with similar results. The values of density of protein bands after normalization to housekeeping were shown. **(F).** Transfection of miR-K6-3p mimic (20 nM) has the same inhibition level on SH3BGR expression as that of KSHV infection. Results shown were from a representative experiment of three independent experiments with similar results. The values of density of protein bands after normalization to housekeeping were shown.

To confirm the specificity of miR-K6-3p targeting of SH3BGR, we first conducted 3'UTR luciferase reporter assay. As shown in Fig 3A, miR-K6-3p inhibited the reporter activity of SH3BGR 3'UTR in a dose-dependent fashion. Western blotting confirmed that miR-K6-3p attenuated the expression of SH3BGR protein in a dose-dependent manner (Fig 3B). We further mutated the putative miR-K6-3p-binding site in the SH3BGR 3'UTR (Fig 3C). Mutation of the putative binding site abolished the inhibitory effect of miR-K6-3p on the SH3BGR 3'UTR reporter activity, while a mutant mimic lacking the seed sequence failed to inhibit the SH3BGR 3'UTR reporter activity (Fig 3D). The mutant mimic designed to match the SH3BGR 3'UTR mutation (Fig 3C) exhibited a strong inhibitory effect on the mutant 3’UTR reporter (Fig 3D). Consistent with these results, a mimic of miR-K6-3p suppressed the expression of endogenous SH3BGR in HUVEC while a mutant mimic of miR-K6-3p lacking the seed sequence did not (Fig 3E). The expression level of SH3BGR in HUVEC transfected with miR-K6-3p mimic was similar to that of KSHV infection (Fig 3F).

**Fig 4:**
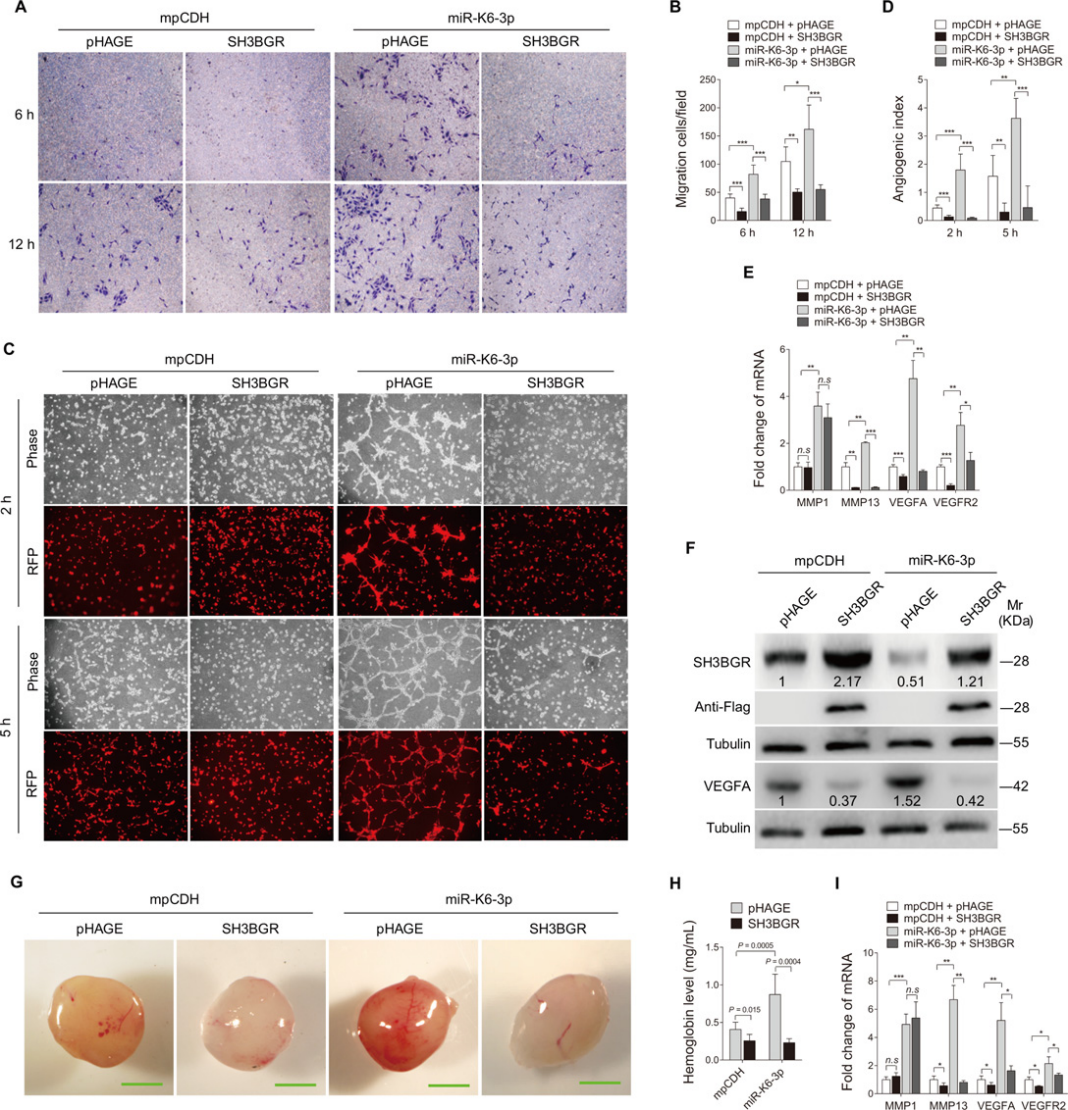
Overexpression of SH3BGR inhibits miR-K6-3p-induced endothelial cell migration and angiogenesis *in vitro* and *in vivo*. **(A)**. Transwell migration assay for HUVEC transduced with lentivirus-mediated miR-K6-3p (**miR-K6-3p**) or empty vector (**mpCDH**), which were subsequently co-transduced with lentivirus-SH3BGR (**SH3BGR**) or its control pHAGE (**pHAGE**), respectively. The representative images were captured at 6 and 12 h post seeding (original magnification, ×100). **(B)**. Quantification of results in **(A)**. The quantified results represent the mean ± SD. Three independent experiments were performed and similar results were obtained, each experiment containing five technical replicates. * *P* < 0.05, ** *P* < 0.01 and *** *P* < 0.001 for Student’s *t*-test. **(C)**. Microtubule formation assay for HUVEC treated as in (**A**). The representative images were captured at 2 and 5 h post seeding (original magnification, ×100). **(D)**. Quantification of results in (**C**). The quantified results represent the mean ± SD. Three independent experiments were performed and similar results were obtained, each experiment containing five technical replicates. ** *P* < 0.01 and *** *P* < 0.001 for Student’s *t*-test. **(E)**. The mRNA expression of MMP1, MMP13, VEGFA and VEGFR2 in HUVEC treated as in (**A**) were determined by qPCR. The quantified results represent the mean ± SD. Three independent experiments were performed and similar results were obtained, each experiment containing four technical replicates. * *P* < 0.05, ** *P* < 0.01 and *** *P* < 0.001 for Student’s *t*-test. *n*.*s*., not significant. **(F)**. Western blotting was performed in HUVEC treated as in (**A**) with the indicated antibodies. The antibody against Flag-tag was used to detect the exogenous expression of SH3BGR. Results shown were from a representative experiment of three independent experiments with similar results. The values of density of protein bands after normalization to housekeeping were shown. **(G)**. HUVEC treated as in (**A**) were examined for their proangiogenic effects in Matrigel plug assay in nude mice as described in the “Materials and Methods” section. Representative photographs of angiogenesis in the nude mice are shown. **(H)**. The hemoglobin level of the Matrigel plugs treated as in (**G**) was determined with hemoglobin content calculated based on the standard curve. Data represent mean ± SD, each group with five tumors (n = 5). Three independent experiments were performed and similar results were obtained. **(I)**. The mRNA expression of MMP1, MMP13, VEGFA and VEGFR2 in the Matrigel plugs treated as in (G) were determined by qPCR. The quantified results represent the mean ± SD. Three independent experiments were performed and similar results were obtained, each experiment containing four technical replicates. * *P* < 0.05, ** *P* < 0.01 and *** *P* < 0.001 for Student’s *t*-test. *n*.*s*., not significant.

We next determined whether SH3BGR mediated miR-K6-3p-induced cell migration and angiogenesis. MiR-K6-3p-expressing HUVEC were transduced with lentivirus-SH3BGR lacking the native 3’UTR sequence and further analyzed for migration and angiogenesis activities. Overexpression of SH3BGR significantly abolished miR-K6-3p-induced migration at 6 and 12 h post-seeding (Fig 4A and 4B). Meanwhile, overexpression of SH3BGR blocked tube formation of HUEVC induced by miR-K6-3p (Fig 4C and 4D). RT-qPCR showed that overexpression of SH3BGR decreased the expression of MMP13, VEGFA and VEGFR2, but not that of MMP1 (Fig 4E). Western-blotting confirmed the suppression of endogenous SH3BGR by miR-K6-3p (Lane 3 *vs* lane 1 in Fig 4F). Transduction with lentivirus-SH3BGR increased the expression level of SH3BGR, and inhibited VEGFA expression (Lanes 2 and 4 in Fig 4F). Furthermore, Matrigel plug assay showed that overexpression of SH3BGR not only inhibited miR-K6-3p-induced angiogenesis (Fig 4G and 4H), but also decreased SMA- and VEGFA-positive cells in plugs induced by miR-K6-3p-transduced cells (S2A and S2B Fig). Consistent with these observations, overexpression of SH3BGR reduced the expression of MMP13, VEGFA and VEGFR2 transcripts in miR-K6-3p-induced plugs (Fig 4I).

### Suppression of SH3BGR Activates the STAT3 Pathway, Which Is Required for miR-K6-3p-Induced Cell Migration and Angiogenesis

**Fig 5:**
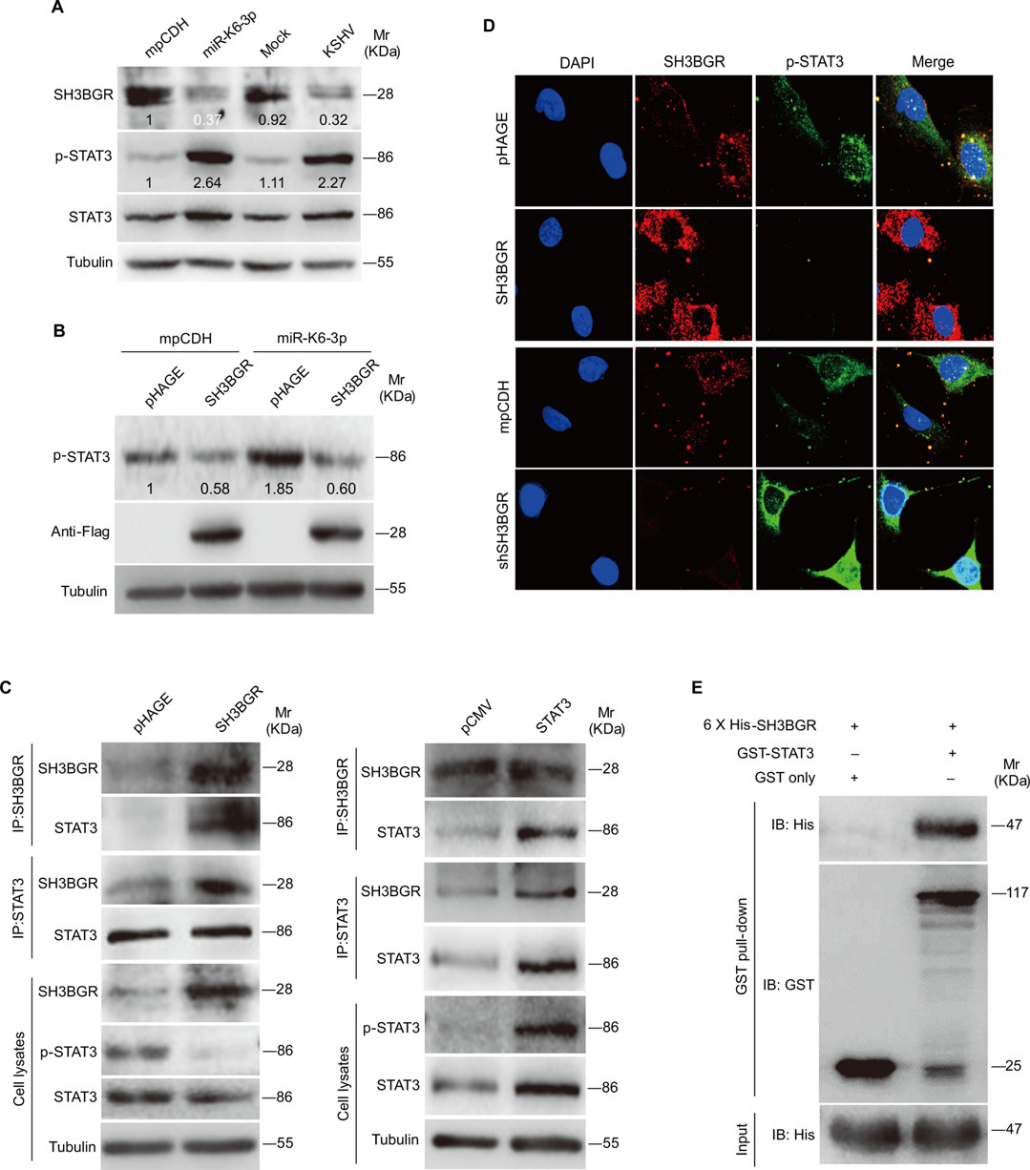
The interaction between SH3BGR and STAT3 in endothelial cells. **(A).** Western blotting analysis of SH3BGR, phosphorylated STAT3 and STAT3 in HUVEC transduced with lentivirus empty vector (**mpCDH**) or lentivirus-miR-K6-3p (**miR-K6-3p**), and HUVEC treated with PBS (**Mock**) or infected with KSHV (**KSHV**), respectively. Results shown were from a representative experiment of three independent experiments with similar results. The values of density of protein bands after normalization to housekeeping were shown. **(B).** Western blotting analysis of phosphorylated STAT3 in HUVEC transduced with lentivirus-mediating empty vector (**mpCDH**) or miR-K6-3p (**miR-K6-3p**), which were subsequently co-transduced with lentivirus SH3BGR (**SH3BGR**) or its control pHAGE (**pHAGE**), respectively. The antibody against Flag-tag was used to detect the exogenous expression of SH3BGR. Results shown were from a representative experiment of three independent experiments with similar results. The values of density of protein bands after normalization to housekeeping were shown. **(C).** The physiological interaction between SH3BGR and STAT3. HUVEC were transduced with lentivirus-SH3BGR (**SH3BGR**) or its control pHAGE (**pHAGE**) and subjected to co-immunoprecipitation with the antibody against SH3BGR (**IP: SH3BGR**) or STAT3 (**IP: STAT3**) followed by Western blotting using indicated antibodies (left panel). Or HUVEC were tranfected with pCMV3-Flag-STAT3 construct (**STAT3**) or its control pCMV3-C-Flag (**pCMV**) and subjected to co-immunoprecipitation with the antibody against SH3BGR (**IP: SH3BGR**) or STAT3 (**IP: STAT3**) followed by Western blotting using indicated antibodies (**right panel**). **(D).** Confocal microscopy for localization of SH3BGR and phosphorylated STAT3. HUVEC transduced with lentivirus SH3BGR (**SH3BGR**) and its control pHAGE (**pHAGE**), or lentivirus-mediated a mixture of short hairpin RNAs targeting SH3BGR (**shSH3BGR**) and its empty vector (**mpCDH**) were stained for SH3BGR (red) and p-STAT3 (green) with indicated antibodies. 4’, 6’-diamidino-2-phenylindole (DAPI) (blue) stains nuclei. **(E).** Association of SH3BGR with full-length STAT3 *in vitro*. GST or GST-STAT3 fusion protein was used to pull down purified 6 x His-SH3BGR protein. Proteins were eluted and SH3BGR bound to GST-STAT3 was detected by immunoblotting with anti-His antibody. Equal loading of purified GST-STAT3 and 6 x His-SH3BGR were detected by immunoblotting with anti-GST antibody (middle panel) and anti-His antibody (bottom panel), respectively.

Since dysregulation of STAT3 is detected in many human cancers including KSHV-infected PEL and KS tumors [53–55], we asked whether STAT3 signaling was involved in miR-K6-3p induction of endothelial cell migration and angiogenesis. In agreement with the previous reports [53, 56, 57], HUVEC cells latently infected by KSHV had increased levels of STAT3 phosphorylation. Importantly, expression of miR-K6-3p alone in endothelial cells was sufficient to increase STAT3 phosphorylation (Fig 5A). Interestingly, there was a negative correlation between the expression level of SH3BGR and STAT3 phosphorylation (Fig 5A). Overexpression of SH3BGR in miR-K6-3p-expressing HUVEC inhibited STAT3 activation (Fig 5B), indicating that SH3BGR negatively regulated the STAT3 activity.

To determine the mechanism of SH3BGR negative regulation of STAT3 activation, we performed co-immunoprecipitation and found that SH3BGR interacted with STAT3 (S3A and S3B Fig and Fig 5C). Overexpression of SH3BGR increased the amount of SH3BGR-immunoprecipiated STAT3 and reduced the level of activated STAT3 (Fig 5C). Similar interaction of SH3BGR and STAT3 was also observed in STAT3-transfected HUVEC cells (Fig 5C). Consistently, overexpression of SH3BGR reduced the level of activated STAT3 in the cytosol and nucleus with simultaneous colocalization of SH3BGR and STAT3 detected by confocal microscope (Fig 5D and S3C Fig). In addition, knockdown of SH3BGR with shRNAs was sufficient to increase the phosphorylated STAT3 level (Fig 5D and S3C Fig). To examine whether SH3BGR may directly associate with STAT3, GST pull-down assays were performed. As shown in Fig 5E, GST-STAT3 was robustly associated with His-SH3BGR, whereas His-SH3BGR was not detected in the GST control pull-down complex. These data indicated that SH3BGR was directly associated STAT3 to inhibit its activation, and miR-K6-3p direct repression of SH3BGR relieved its inhibition of STAT3 phosphorylation, resulting in higher level of STAT3 activation.

**Fig 6:**
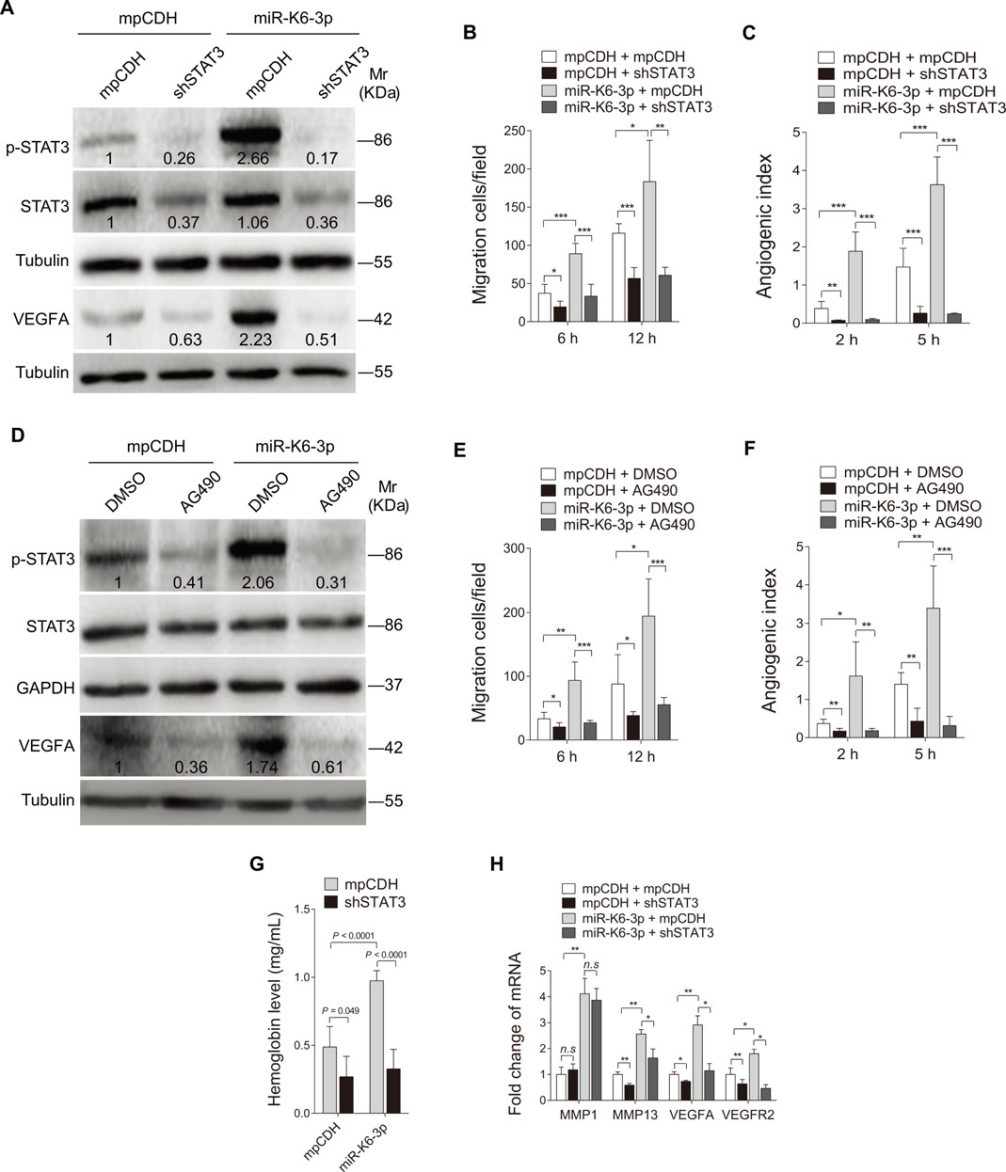
Activation of STAT3, which was negatively regulated by SH3BGR, contributes to miR-K6-3p-induced endothelial cell migration and angiogenesis. **(A)**. Western blotting analysis of phosphorylated STAT3 and VEGFA in HUVEC transduced with lentivirus-mediated empty vector (**mpCDH**) or miR-K6-3p (**miR-K6-3p**), and further transduced with lentivirus-mediated a mixture of short hairpin RNAs targeting STAT3 (**shSTAT3**). Results shown were from a representative experiment of three independent experiments with similar results. The values of density of protein bands after normalization to housekeeping were shown. **(B)**. Transwell migration assay for HUVEC treated as in (**A**). The quantified results represent the mean ± SD. Three independent experiments were performed and similar results were obtained, each experiment containing five technical replicates. * *P* < 0.05, ** *P* < 0.01 and *** *P* < 0.001 for Student’s *t*-test. (C). Microtubule formation assay for HUVEC treated as in (A). The quantified results represent the mean ± SD. Three independent experiments were performed and similar results were obtained, each experiment containing five technical replicates. ** *P* < 0.01 and *** *P* < 0.001 for Student’s *t*-test. **(D)**. Western blotting analysis of phosphorylated STAT3, STAT3 and VEGFA in HUVEC transduced with lentivirus-mediated empty vector (**mpCDH**) or miR-K6-3p (**miR-K6-3p**) and further treated with the JAK2 inhibitor, AG490 (**AG490**) or its control (**DMSO**). Results shown were from a representative experiment of three independent experiments with similar results. The values of density of protein bands after normalization to housekeeping were shown. **(E)**. Transwell migration assay for HUVEC treated as in (**D**). The quantified results represent the mean ± SD. Three independent experiments were performed and similar results were obtained, each experiment containing five technical replicates. * *P* < 0.05, ** *P* < 0.01 and *** *P* < 0.001 for Student’s *t*-test. **(F)**. Microtubule formation assay for HUVEC treated as in (D). The quantified results represent the mean ± SD. Three independent experiments were performed and similar results were obtained, each experiment containing five technical replicates. * *P* < 0.05, ** *P* < 0.01 and *** *P* < 0.001 for Student’s *t*-test. **(G)**. HUVEC treated as in (**A**) were examined for their proangiogenic effects in Matrigel plug assay in nude mice as described in the “Materials and Methods” section. The hemoglobin levels of the Matrigel plugs were determined with hemoglobin content calculated based on the standard curve. Data represent mean ± SD, each group with five tumors (n = 5). Three independent experiments were performed and similar results were obtained. **(H)**. The mRNA expression of MMP1, MMP13, VEGFA and VEGFR2 in the Matrigel plugs treated as in (G) were determined by qPCR. The quantified results represent the mean ± SD. Three independent experiments were performed and similar results were obtained, each experiment containing four technical replicates. * *P* < 0.05, ** *P* < 0.01 and *** *P* < 0.001 for Student’s *t*-test. *n*.*s*., not significant.

To determine whether STAT3 mediates miR-K6-3p-induced cell migration and angiogenesis, we transduced miR-K6-3p-expressing HUVEC with a mixture of shRNAs to knock down STAT3 expression (shSTAT3; Fig 6A and S4 Fig). Transwell migration and microtubule formation assays showed that knockdown of STAT3 inhibited miR-K6-3p-induced migration and tube formation (Fig 6B and 6C). To further confirm these observations, AG490, an inhibitor of JAK2/STAT3 pathway, was used to treat miR-K6-3p-transduced HUVEC. AG490 not only decreased the level of phosphorylated STAT3 (Fig 6D) but also inhibited cell migration and tube formation (Fig 6E and 6F). Furthermore, Matrigel plug assay showed that knock-down of STAT3 by shSTAT3 inhibited miR-K6-3p-induced angiogenesis (Fig 6G). Knockdown of STAT3 *in vivo* also blocked miR-K6-3p induction of MMP13, VEGFA, and VEGFR2 (Fig 6H).

Together these data indicated that miR-K6-3p-induced cell migration and angiogenesis is mediated by activating the STAT3 pathway.

### Deletion of miR-K6 from the KSHV Genome Attenuates KSHV-Induced Cell Migration and Angiogenesis

**Fig 7:**
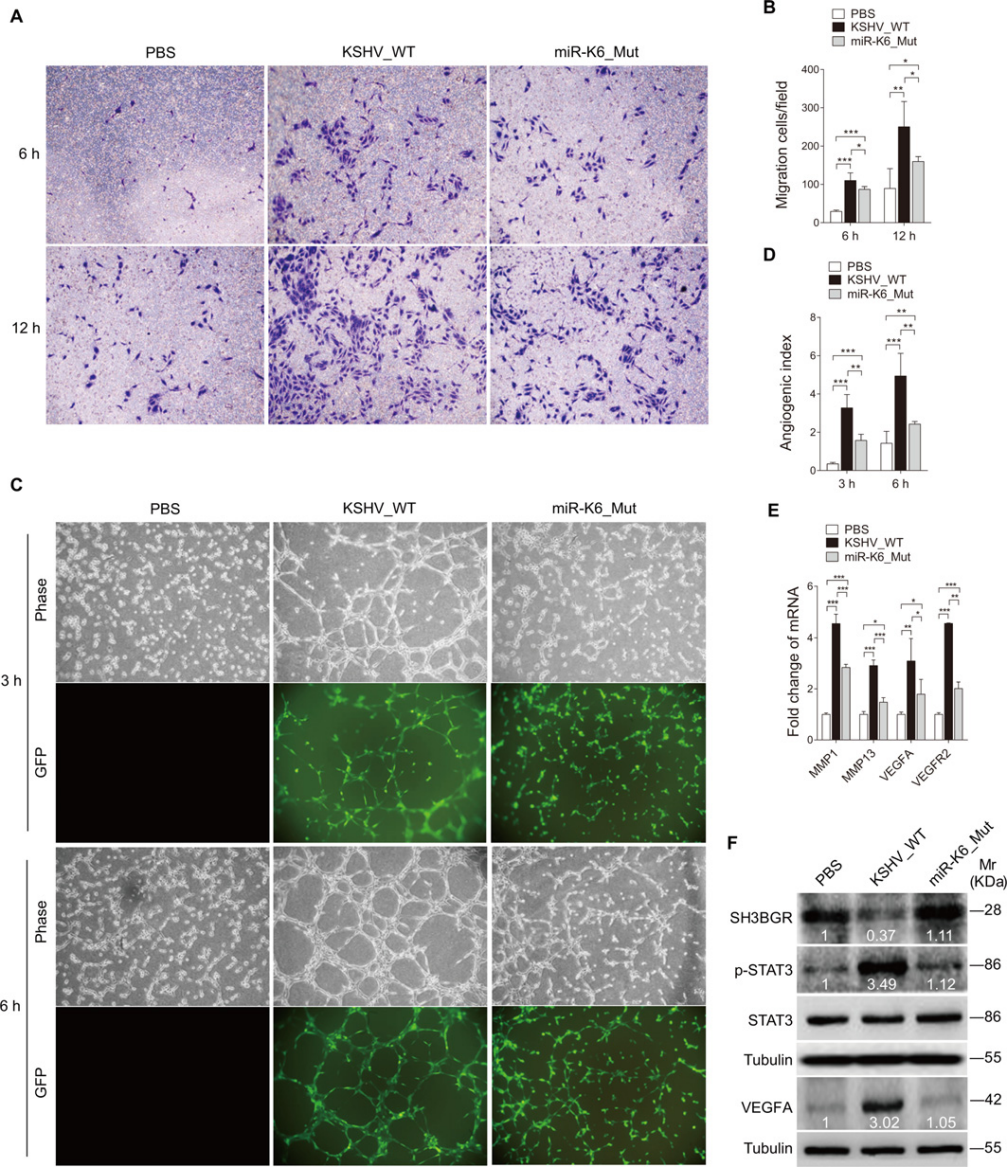
Deletion of miR-K6 from KSHV genome attenuates KSHV induction of endothelial cell migration and angiogenesis. **(A)**. Transwell migration assay for HUVEC were treated with PBS (PBS), infected with BAC16 KSHV wide type virus (KSHV_WT) or BAC16 KSHV miR-K6 deletion mutant virus (miR-K6_Mut). The representative images were captured at 6 and 12 h post seeding (original magnification, ×100). **(B)**. Quantification of the results in (**A**). The quantified results represent the mean ± SD. Three independent experiments were performed and similar results were obtained, each experiment containing five technical replicates. * *P* < 0.05, ** *P* < 0.01 and *** *P* < 0.001 for Student’s *t*-test. (**C**). Microtubule formation assay for HUVEC treated as in (**A**). The representative images were captured at 3 and 6 h post seeding (original magnification, ×100). **(D)**. Quantification of the results in (**C**). The quantified results represent the mean ± SD. Three independent experiments were performed and similar results were obtained, each experiment containing five technical replicates. ** *P* < 0.01 and *** *P* < 0.001 for Student’s *t*-test. **(E)**. The mRNA expression of MMP1, MMP13, VEGFA and VEGFR2 in HUVEC treated as in (**A**) were determined by RT-qPCR. The quantified results represent the mean ± SD. Three independent experiments were performed and similar results were obtained, each experiment containing four technical replicates. * *P* < 0.05, ** *P* < 0.01 and *** *P* < 0.001 for Student’s *t*-test. **(F)**. Western blotting analysis of expression of SH3BGR, phosphorylated STAT3, STAT3 and VEGFA in HUVEC treated as in (**A**) with the indicated antibodies. Results shown were from a representative experiment of three independent experiments with similar results. The values of density of protein bands after normalization to housekeeping were shown.

To further confirm that miR-K6-3p induced endothelial cell migration and angiogenesis by targeting the SH3BGR/STAT3 pathway, we infected HUVEC and obtained latent cultures of a BAC16 miR-K6_mut virus with miR-K6 deleted from the KSHV genome. RT-qPCR did not detect the expression of miR-K6 in cells infected with the miR-K6_mut virus while the expression of other miRNAs was not affected (S5A Fig). The levels of LANA in miR-K6-mut-infected cells were similar to that of cells infected by the wild type (WT) KSHV (S5B Fig). As shown in Fig 7A and 7B, the level of cell migration in HUVEC infected by the mutant virus was significantly lower than that of cells infected by the WT virus albeit it remained higher than that of uninfected cells. Similarly, deletion of miR-K6 decreased the level of KSHV-induced tube formation in HUVEC (Fig 7C and 7D). Mutant cells remained to have higher angiogenesis activity than that of uninfected cells. Consistent with these observations, cells infected by the mutant virus had decreased levels of MMP1, MMP13, VEGFA, and VEGFR2 mRNAs than those infected by the WT virus (Fig 7E). Importantly, cells infected by the mutant virus had SH3BGR expression at level similar to mock infected cells. However, cells infected by the mutant virus reduced STAT3 phosphorylation and VEGFA expression compared to cells infected by the WT virus (Fig 7F).

Considering that deletion of miR-K6 abolished the expression of both miR-K6-3p and miR-K6-5p, to show that the observed phenotypes were due to the absence of miR-K6-3p from the KSHV genome, we transfected WT KSHV-infected HUVEC with a specific miR-K6-3p inhibitor. As expected, inhibition of miR-K6-3p was sufficient to reduce cell migration and angiogenesis in WT KSHV-infected HUVEC (S6 Fig and Fig 6).

**Fig 8:**
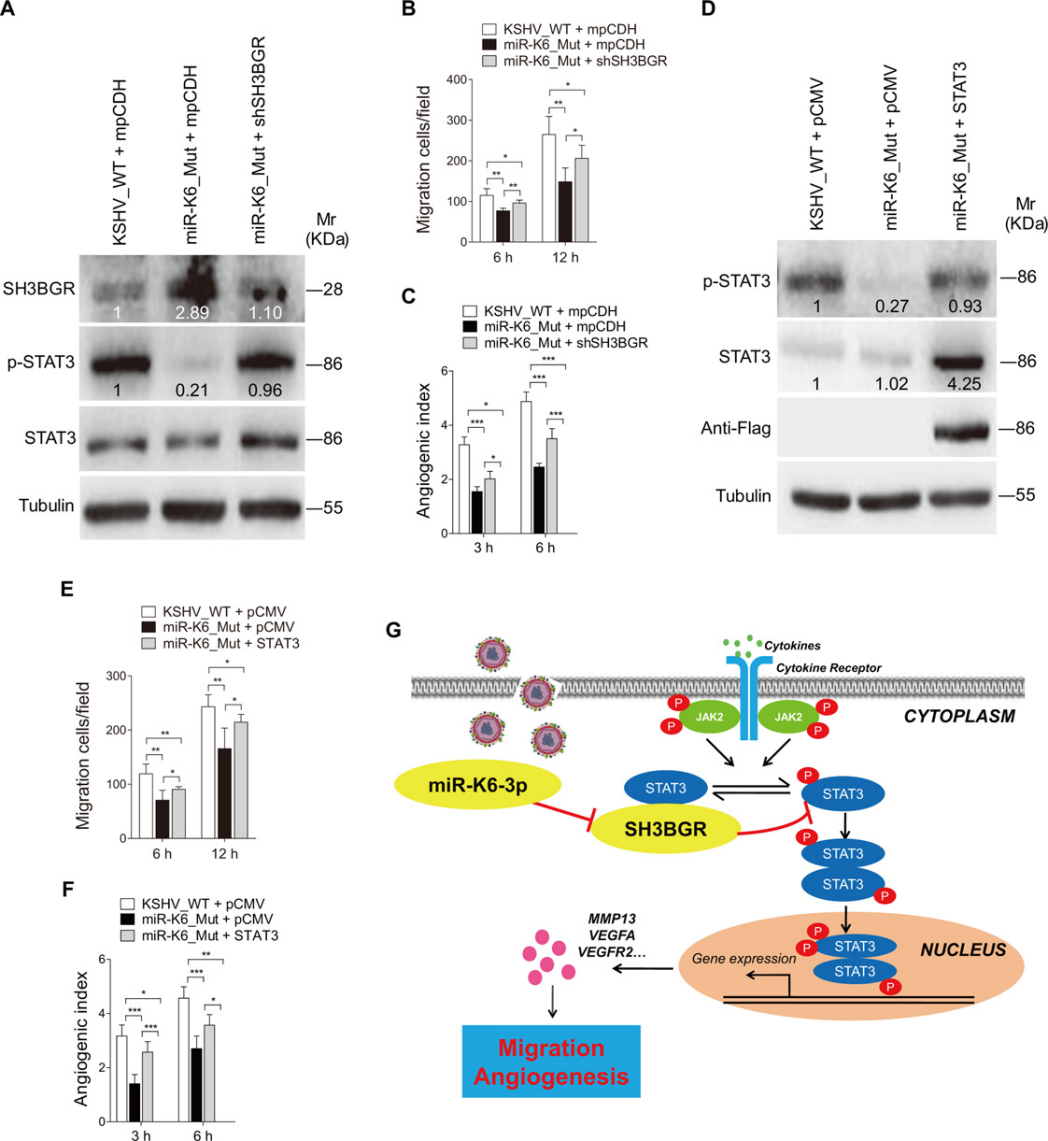
SH3BGR/STAT3 signaling partially mediates miR-K6-induced endothelial cell migration and angiogenesis. **(A)**. Western blotting analysis of expression of SH3BGR, phosphorylated STAT3 and STAT3 in HUVEC infected with BAC16 KSHV wide type virus (**KSHV_WT**) or BAC16 KSHV miR-K6 deletion mutant virus (**miR-K6_Mut**) and further transduced with lentivirus-mediated a mixture of short hairpin RNAs targeting SH3BGR (**shSH3BGR**). Results shown were from a representative experiment of three independent experiments with similar results. The values of density of protein bands after normalization to housekeeping were shown. **(B)**. Transwell migration assay for HUVEC treated as in (**A**). The quantified results represent the mean ± SD. Three independent experiments were performed and similar results were obtained, each experiment containing five technical replicates. * *P* < 0.05 and ** *P* < 0.01 for Student’s *t*-test. **(C)**. Microtubule formation assay for HUVEC treated as in (**A**). The quantified results represent the mean ± SD. Three independent experiments were performed and similar results were obtained, each experiment containing five technical replicates. * *P* < 0.05 and *** *P* < 0.001 for Student’s *t*-test. **(D)**. Western blotting analysis of expression of phosphorylated STAT3 and STAT3 in HUVEC infected with BAC16 KSHV wide type virus (**KSHV_WT**) or BAC16 KSHV miR-K6 deletion mutant virus (**miR-K6_Mut**) and further tranfected with pCMV3-Flag-STAT3 construct (**STAT3**) or its control pCMV3-C-Flag (**pCMV**). The antibody against Flag-tag was used to detect the exogenous expression of STAT3. Results shown were from a representative experiment of three independent experiments with similar results. The values of density of protein bands after normalization to housekeeping were shown. **(E)**. Transwell migration assay for HUVEC treated as in (**D**). The quantified results represent the mean ± SD. Three independent experiments were performed and similar results were obtained, each experiment containing five technical replicates. * *P* < 0.05 and ** *P* < 0.01 for Student’s *t*-test. **(F)**. Microtubule formation assay for HUVEC treated as in (**D**). The quantified results represent the mean ± SD. Three independent experiments were performed and similar results were obtained, each experiment containing five technical replicates. * *P* < 0.05, ** *P* < 0.01, and *** *P* < 0.001 for Student’s *t*-test. **(G)**. Schematic representation of the mechanism by which miR-K6-3p facilitates endothelial cell migration and angiogenesis. In normal endothelial cells, SH3BGR binds STAT3 to inhibit STAT3 activation. During KSHV infection, reduction of SH3BGR as a result of miR-K6-3p targeting releases STAT3 to activate STAT3, leading to the higher expression level of matrix metalloproteinases (MMPs) and angiogenic factors, and ultimately promotes endothelial cell migration and angiogenesis.

To further confirm the observations, we performed knock-down of SH3BGR with a mixture of shRNAs (shSH3BGR) in mutant cells. As expected, knock-down of SH3BGR in miR-K6_mut-infected HUVEC with shSH3BGRs increased the level of activated STAT3 compared to cells transduced with the control vector (Fig 8A and S7 Fig). Meanwhile, knock-down of SH3BGR partially increased cell migration and angiogenesis of the cells infected by miR-K6_mut virus (Fig 8B and 8C). Similarly, overexpression of STAT3 in miR-K6_mut-infected cells increased cell migration and angiogenesis (Fig 8D, 8E and 8F). Similar increase in cell migration and angiogenesis was also observed in the mutant cells following overexpression of miR-K6-3p (S8 Fig). Together these results show that, in the context of KSHV infection, miR-K6 activates the STAT3 pathway to promote cell migration and angiogenesis by targeting SH3BGR.

## Discussion

SH3BGR, a protein of 239 amino acids, contains a conserved N-terminal region, and a less conserved C-terminal region highly enriched in glutamic acid residues [58]. The N-terminal region contains a proline-rich sequence (PLPPQIF), which conforms both the SH3 binding motif (PXXP) [59] and the Homer EVH1 binding motif (PPXXF) [60]. SH3BGR is involved in the pathogenesis of Down syndrome congenital heart disease and obesity [49, 50]. However, whether dysregulation of SH3BGR expression is associated with the development and progression of tumors, including KS, remains unknown. In this study, we detected the expression of SH3BGR in the skin and endothelial cells. We revealed that SH3BGR suppresses cell migration and angiogenesis by interacting and inhibiting STAT3 activation. Furthermore, KSHV miR-K6-3p directly targets SH3BGR to activate the STAT3 pathway, resulting in the enhanced cell migration and angiogenesis.

STAT3 is constitutively active in a significant proportion of human solid tumors and regulates a number of important functions in tumorigenesis, including cell cycle progression, apoptosis, tumor angiogenesis, invasion and metastasis, and tumor cell evasion of immune system [54, 55, 61–64]. Recent studies suggest that persistent activation of STAT3 also plays a critical role in KSHV-associated tumors [65, 66]. For instance, KSHV latent infection in primary endothelial cells resulted in aberrant and chronic activation of STAT3, and activation of STAT3 enhanced KSHV latency [57, 67]. STAT3 signaling was shown to induce the pro-survival protein survivin, which was found to be an important factor in STAT3-mediated pro-survival effects in PEL cells [53]. De novo KSHV infection resulted in lymphatic endothelial cell reprogramming of blood endothelial cells by activating STAT3 in a gp130-dependent but vIL-6-independent manner [68]. Therefore, STAT3 signaling, in part, mediated via gp130, is important for the maintenance of KSHV latency and for the survival of latently infected cells. Here, we have demonstrated that ectopic expression of miR-K6-3p in HUVEC led to the increased STAT3 signaling while deletion of miR-K6 from the KSHV genome significantly decreased the phosphorylation level of STAT3 in KSHV-infected endothelial cells. Thus, miR-K6-3p contributes to aberrant STAT3 signaling during KSHV infection. Further, our co-immunoprecipitation and GST-pull down results indicated that SH3BGR physiologically interacted with STAT3 and inhibited STAT3 activation in normal endothelial cells. However, when endothelial cells were infected by KSHV, the expression of miR-K6-3p resulted in the downregulation of SH3BGR, which released STAT3 from SH3BGR inhibition and activated STAT3. The outcomes were enhanced cell migration and angiogenesis. Thus, our results revealed a novel role of STAT3 signaling in the metastasis and angiogenesis of KSHV-related tumors.

De novo KSHV infection of human endothelial cells results in an increased secretion of several growth factors, cytokines, chemokines, and angiogenic factors, including MMPs and VEGF, which are involved in cell motility [69, 70] and angiogenesis [71], respectively. Indeed, several MMPs have been reported to contribute to KS spindle cell migration and invasion [4–7]. Consistently, in the current study, MMP1 and MMP13 were significantly increased following ectopic expression of miR-K6-3p. Inversely, deletion of miR-K6 from the KSHV genome resulted in the reduced induction of MMPs by KSHV in endothelial cells. However, either overexpression of SH3BGR or knock-down of STAT3 did not change MMP1 level, indicating that miR-K6-3p may target the other signaling pathway rather than the SH3BGR/STAT3 axis to regulate MMP1.

Our results also showed that deletion of miR-K6 from the KSHV genome did not completely abolish the capabilities of migration and angiogenesis of KSHV-infected endothelial cells. This is more likely to reflect the fact that, besides miR-K6, KSHV encodes a number of proteins and miRNAs that may also contribute to tumor angiogenesis and metastasis. While miR-K6 was removed from the KSHV genome, other viral products could still exert their functions to regulate cell migration and angiogenesis. For example, KSHV-encoded latent nuclear antigen (LANA), G protein-coupled receptor (vGPCR), interleukin-6 (vIL-6), ORF-K1, and ORF-K15 have been shown to regulate cell migration and angiogenesis [72–79]. Similarly, our recent study also indicated that miR-K3 promotes endothelial cell migration and invasion [44].

Interestingly, either inhibition of SH3BGR or overexpression of STAT3 did not totally reverse KSHV-induced cell migration and angiogenesis. These results imply that, besides the SH3BGR/STAT3 pathway, miR-K6-3p might target the other cellular genes to induce cell migration and angiogenesis. In addition, both miR-K6-5p and miR-K6-3p were derived from the same precursor miRNA and miR-K6-3p might also be involved in KSHV-induced angiogenesis [40].

In summary, we revealed that miR-K6-3p promotes endothelial cell migration and angiogenesis by targeting the SH3BGR/STAT3 pathway (Fig 8G). These results constitute parts of the novel regulatory networks between KSHV miRNAs and their multiple targets. Considering the multiple functions and targets of viral miRNAs, further studies will be required to explore the molecular mechanisms by which the other KSHV miRNAs contribute to KSHV-induced malignancies.

## Materials and Methods

### Ethics Statement

The clinical section of the research was reviewed and ethically approved by the Institutional Ethics Committee of the First Affiliated Hospital of Nanjing Medical University (Nanjing, China; Study protocol # 2015-SR-116). Written informed consent was obtained from all participants, and all samples were anonymized. All participants were adults.

The animal experiments were approved by the Institutional Animal Care and Use Committee of Nanjing Medical University (Animal protocol # NJMU/IACUC_2013-8-18-01). All animal care and use protocols were performed in accordance with the Regulations for the Administration of Affairs Concerning Experimental Animals approved by the State Council of People's Republic of China.

### Cell Culture

Primary human umbilical vein endothelial cells (HUVEC) were isolated from freshly obtained human umbilical cords by digesting the interior of the umbilical vein with collagenase (Sigma, St. Louis, MO, USA) and cultured in complete EBM-2 culture media (LONZA, Allendale, NJ, USA) [80]. HUVEC were used between passage 3 and 6. HEK293T were cultured as previously described [81]. ISLK-BAC16 cells and iSLK-BAC16△miR-K6 cells were maintained as previously described [82]. All cells were cultured at 37°C in a humidified, 5% CO_2_ atmosphere.

### Plasmids

The construct pGL3-SH3BGR 3’UTR and the mutant SH3BGR 3'UTR construct were generated by cloning the full length of the SH3BGR 3’UTR sequence and mutant 3’UTR sequence into the downstream of the luciferase sequence in the pGL3-Control plasmid (Promega, Shanghai, China), respectively. Human SH3BGR gene with a Flag tag at the C-terminus was amplified using the cDNA of HUVEC as PCR templates and inserted into the lentiviral transferring plasmid pHAGE to generate recombinant pHAGE-SH3BGR as previously described [78, 83]. The miRNA (miR-K6-3p) expressing plasmid was constructed by two steps: First, the miR-30 precursor stem-loops plus RFP coding sequences were amplified from the pTRIPZ plasmid (Open Biosystems, AL, USA) and inserted into another lentiviral plasmid pCDH-CMV-MCS-EF1-copGFP (System Bioscience, CA, USA) to create a new lentiviral plasmid, which has GFP and RFP two signals and was designed as modified pCDH (mpCDH) [44]. Second, the precursor stem-loops of miR-K6-3p was amplified using primers (forward) 5’-CAG AAG GCT CGA GAA GGT ATA TTG CTG TTG ACA GTG AGC G-3’ and (reverse) 5’-CTA AAG TAG CCC CTT GAA TTC CGA GGC AGT AGG CA-3’ and cloned into mpCDH. Meanwhile, the mpCDH plasmid was also used for the short hairpin RNA (shRNA) expressing lentiviral vector. ShRNA complementary sequences to SH3BGR and STAT3 were listed in Table 3. The pCMV3-Flag-STAT3 construct containing STAT3 cDNA was purchased from Sino Biological Inc. (Beijing, China). The GST-STAT3 plasmid was constructed by inserting the full-length STAT3 open reading frame into pGEX-4T-3 (GE Healthcare, Piscataway, USA). Plasmid expressing SH3BGR fusion protein was constructed in the pET32a(+) vector (EMD Chemicals, CA, USA). In this study, the control of pHAGE-SH3BGR was named as pHAGE, and the controls of miR-K6-3p and all the shRNA were a modified pCDH (mpCDH for short).

**Table 3:** The sequences of the shRNAs.

Gene	shRNA No.	Sequence of shRNA (5’to 3’)
STAT3	sh1	GCAACAGAUUGCCUGCAUUTT
	sh2	CCCGUCAACAAAUUAAGAATT
	sh3	GCGUCCAGUUCACUACUAATT
SH3BGR	sh1	GGAGGUGGAUGAGAGAGAATT
	sh2	GGGAUCAGAGAAGGCUGAATT
	sh3	GGAGAAGAAUGAAGAAGAATT
	sh4	GGAGAAGACGAAGAUUCCUTT

### Transfection and Luciferase Reporter Assay

Transfection of HUVEC was performed with the Effectence transfection reagent (Qiagen, Valencia, CA, USA), while the other transfections were performed with Lipofectamine 2000 (Invitrogen, Carlsbad, CA, USA) following the manufacturer’s instructions. For luciferase assay, HEK293T cells (1×10^5^) were co-transfected with miRNA mimic, luciferase reporter DNA and Renilla vector pRL-TK (Promega, Madison, WI), and then harvested at 24 h post-transfection. Relative luciferase activity was assayed using the Promega dual-luciferase reporter assay system. Firefly activity was normalized to internal Renilla luciferase levels.

### Antibodies, Western Blotting and Reagents

Anti-KSHV LANA rat monoclonal antibody (MAb) was purchased from Advanced Biotechnologies Inc.(Columbia, MD, USA) [81]. Anti-phospho-STAT3 (Y705) rabbit MAb, anti-STAT3 mouse polyclonal antibody (PAb), and anti-Flag M2 rabbit MAb were obtained from Cell Signaling Technologies (Beverly, MA, USA). Anti-SH3BGR mouse MAb, anti-GAPDH mouse MAb, anti-α-Tubulin mouse MAb, and horseradish peroxidase (HRP)-conjugated goat anti-mouse or anti-rabbit IgG were all purchased from Santa Cruz Biotechnology (Santa Cruz, CA, USA). Anti-His mouse MAb, anti-mouse immunoglobulin G (IgG) and anti-GST mouse MAb were from Beyotime Institute of Biotechnology (Nantong, Jiangsu, China). Western blotting analysis was performed as described previously [84, 85]. AG490, a JAK2 inhibitor, was purchased from Selleck Chemicals (Shanghai, China).

### BAC16 and miR-K6 Mutant BAC16△miR-K6

Wild type recombinant KSHV BAC16 and a KSHV mutant with miR-K6 deleted, BAC16△miR-K6, were previously described [24, 40, 82]

### Production of BAC16 Virus Stock

Production of KSHV BAC16 virus was performed according to the previous study [44].

### Production and Transduction of Lentivirus

To obtain the recombinant lentivirus, the virus-packaging cells HEK293T were seeded for 24 h later and then co-transfected with lentiviral plasmids, packaging vector psPAX2 and envelope vector pMD2.G as previously described [86]. The virus containing supernatants were collected 48 h after transfection.

### Virus Titration

Ten-fold serial dilution of lentivirus was prepared in PBS from 10^−1^ to 10^−10^. The HUVEC cells at 50% confluence was prepared in a 48-well culture plate. A 100 μl of each virus dilution was added in each well. The plate was incubated at 37°C for 3 h to allow adsorption. Then 100 μl of maintenance medium was added in each well and incubated at 37°C in 5% CO2 for 48 h. The number of fluorescent cells in the lowest dilution of virus was considered as an end point to calculate the virus titer.

### Transwell Migration Assay

Transwell migration assay was performed as previously described [44]. The number of migrated cells was determined by counting stained cells and the average cell number per field for each well was calculated. The counting was blinded by three individuals, including one who was blinded to the results. For each experiment, at least four replicate wells were used and the representative images were taken from five randomly selected fields of each well.

### RNA Isolation and Real-Time Quantitative Reverse Transcription-PCR (qPCR)

Total RNA was isolated from cells by Trizol reagent (Invitrogen, Carlsbad, CA, USA) and subjected to the Promega Reverse-Transcription Kit (Promega, Madison, WI) to obtain cDNA. The sequences of specific primers of RT-qPCR for several genes were listed in Table 4. Quantitative PCR (qPCR) was performed using SYBR *Premix Ex Taq* Kit (TaKaRa Biotechnology Co. Ltd., Dalian, China) according to the manufacturer’s instructions.

**Table 4:** The sequences of specific primers for qPCR.

Target	Application	Primer
MMP1	RT-qPCR	F: 5′- AATGTGCTACACGGATACCC -3′
		R: 5′- CTTTGTGGCCAATTCCAGGA -3′
MMP13	RT-qPCR	F: 5′-TGGAAGGATGCCTTTTTTTCTC -3′
		R: 5′- CACCCTCCCCAAGTATCAATAGG -3′
VEGFA	RT-qPCR	F: 5′-CTTGCCTTGCTGCTCTACCT -3′
		R: 5′-GATTCTGCCCTCCTCCTTCT -3′
VEGFR2	RT-qPCR	F: 5′- TGGGAACCGGAACCTCACTATC -3′
		R: 5′- GTCTTTTCCTGGGCACCTTCTATT -3′
LANA	RT-qPCR	F: 5′- CCG AGG ACG AAA TGG AAGTG -3′
		R: 5′-GGTGATGTTCTGAGTACATAGCGG -3′
β-actin	RT-qPCR	F: 5′- TTGCCGACAGGATGCAGAAGG A -3′
		R: 5′- AGGTGGACAGCGAGGCCAGGAT -3′

### Sample Preparation, TMT Labeling, Mass Spectrometry, and Data Analysis

Mass spectrometry analysis was done as previously described [87]. Briefly, proteins were reduced, alkylated, and digested with trypsin. The resulting peptides were labeled with isobaric tandem mass tags (TMT 6-plex; Thermo Fisher Scientific Inc., San Jose, CA), mixed, and fractionated by strongcation exchange chromatography. Peptide digests were analyzed by nanoscalereversed phase liquid chromatography (Easy-nLC, Thermo Fisher Scientific Inc.) coupled online with an LTQ-OrbitrapVelos mass spectrometer (Thermo Fisher Scientific Inc.). Spectra were searched using Maxquant (version 1.2.2.5), and results were filtered to 1% FDR at the unique peptide level using the COMPASS software suite.

### Immunohistochemistry (IHC)

The KS clinical tissue specimens and the normal skin tissue specimens were provided by the First Affiliated Hospital of Nanjing Medical University for hematoxylin and eosin (H&E) and immunohistochemistry (IHC) staining. All the samples were formalin-fixed, parafin-embedded, and immunostained with the indicated antibodies as previously described [44, 86]. The results were processed and analyzed using Image-Pro Plus 6.0 image analysis system (Media Cybernetics, Silver Spring, MD). Five random fields were chosen under the microscope and further measured for area and intensity of the expression of target protein, with the expression level of target protein calculated based on average absorbance (gray).

### Matrigel Plug Assay for Angiogenesis in Nude Mice

Matrigel plug assay was performed as previously described [78, 86, 88]. Male athymic BALB/c nu/nu mice of 3-4-week-old (Nanjing Biomedical Research Institute of Nanjing University, Nanjing, China) were maintained under pathogen-free conditions. The cells were harvested at subconfluence, washed with phosphate-buffered saline and resuspended in serum-free medium. Cell aliquots (0.2 ml) were mixed with 0.4 ml of High Concentration Matrigel (BD Biosciences, Bedford, MA, USA), and the mixture was immediately injected subcutaneously into the right flanks of nude mice. The mice were killed 10 days after the injection, and the Matrigel plugs were removed from the mice. The hemoglobin content of the Matrigel was determined using Drabkin’s reagent kit (Sigma-Aldrich) according to the manufacturer’s instructions. The final hemoglobin concentration was calculated from a standard calibration curve after spectrophotometric analysis at 540 nm.

### Microtubule Formation Assay

The microtubule formation assay was performed on micro-slide Angiogenesis ibiTreat (ibidi, Martinsried, Germany) coated with 10 ul of Matrigel (BD Biosciences) as previously described [88]. HUVEC was seeded at 5 x 10^3^ cells per well with 50 μl serum-free basic medium. Tubule formation was quantified by counting the number of branching points and measuring the total length of the capillary tubes in at least three images using NIH Image software.

### Co-Immunoprecipitation

Immunoprecipitation was performed using a standard protocol. Briefly, cells were collected, rinsed twice with cold PBS, and lysed in Lysis/Wash buffer (150 mM NaCl, 1 mM EDTA, 5% glycerol, 1% NP-40, 25 mM Tris-HCl, pH7.4) supplemented with protease inhibitors, phosphatase inhibitors and phenylmethylsulfonyl fluoride (PMSF). Lysates were cleared by centrifugation at 11,000 g at 4°C for 10 min. Supernatants were incubated with 5 μg of the specified antibody overnight at 4°C followed by 50 μl of protein A-beads for 4 h at 4°C with gentle rotation. The beads were then pelleted at 5, 000 g for 2 min and washed 3 times in 1 ml ice-cold Lysis/Wash buffer containing 1mM PMSF and 50 g/ml aprotinin. Antibody-protein conjugates were eluted by boiling (5 min) and samples were then subjected to SDS-PAGE and immunoblotting as described above.

### Confocal Microscopy

Immunolocalization was performed as previously described [78]. The stained cells were examined and photographed using a Zeiss Axiovert 200M laser scanning confocal microscope (Carl Zeiss, Freistaat Thuringen, Germany).

### Glutathione S-Transferase (GST) Pull-Down Assay

Purification of GST- or His-tagged proteins and GST pull-down assay were performed as previously described [89]. Briefly, GST fusion proteins were purified from bacteria using GST-BindTM Resin (Novagen, Darmstadt, Germany) according to the manufacturer’s protocol and resuspended in PBS containing 0.5% Nonidet P-40 and protease inhibitors. Equal amounts of GST or GST-STAT3 fusion protein were incubated with purified SH3BGR protein (His-tagged) for 2 h at 4 ℃ in binding buffer (50 mM Tris-HCl, PH 7.5, 100 mM NaCl, 0.25% Triton-X100, 35 mM 2-Me). Proteins were eluted and SH3BGR binding to GST-STAT3 fusion protein was then detected by immunoblotting with antibody against His.

### Statistical Analysis

Quantitative data were presented as mean ± SD Two-sided Student’s *t*-test was used to determine the significance between different treatment groups. *P* < 0.05 was considered statistically significant. All the experiments were repeated three times, unless otherwise stated.

## Supporting Information

S1 Table A list of accession numbers/ID numbers for genes mentioned in the text. (DOCX)

S2 Table A list of accession numbers/ID numbers for miRNAs mentioned in the text. (DOCX)

S1 Fig Determination of transduction efficiency of lentivirus-mediated miR-K6-3p in endothelial cells. **(A)**. KSHV miR-K6-3p expression in HUVEC infected with KSHV BAC16 virus induced from iSLK-BAC16 cells or transduced by the different MOI of lentiviral miR-K6-3p were determined by RT-qPCR. The miR-K6-3p level in KSHV group was set as ‘‘1” for comparison. The quantified results represent the mean ± SD. Three independent experiments were performed and similar results were obtained, each experiment containing four technical replicates. **(B)**. HUVEC were transduced with 1 MOI lentivirus empty vector (mpCDH; top) and lentivirus-miR-K6-3p (miR-K6-3p; bottom), and representative images were taken under the light microscope (Phase; left) and fluorescent microscope (RFP; right) (Original magnification, ×100). **(C)**. HUVEC were transduced with 1 MOI of lentivirus empty vector (mpCDH; left) and lentivirus-miR-K6-3p (miR-K6-3p; right) were analyzed for RFP expression by flow cytometry to determine transduction efficiency. **(D)**. Luciferase activity was detected in 1 MOI of lentivirus empty vector (mpCDH) or lentivirus-miR-K6-3p (miR-K6-3p) transduced HUVEC transfected by the pGL3-Control (Control) or the pGL3-miR-K6-3p sensor reporter (Sensor). *n*.*s*., not significant.
(TIF)

S2 Fig Ectopic expression of SH3BGR inhibits miR-K6-3p-induced angiogenesis. **(A)**. The Matrigel plugs treated as in (**Fig 4G**) were fixed, sectioned, and stained with hematoxylin and eosin (top; original magnification, x400), SMA (middle; original magnification, x400) and VEGFA (bottom; original magnification, x400). **(B)**. Quantification of results in (**A**). ** *P* < 0.01 and *** *P* < 0.001 for Student’s *t*-test.
(TIF)

S3 Fig The physiological interaction between SH3BGR and STAT3 in endothelial cells. **(A)**. HUVEC subjected to co-immunoprecipitation with the antibody against immunoglobulin G (**IgG**) or SH3BGR (**SH3BGR**) followed by Western blotting using indicated antibodies. Results shown were from a representative experiment of three independent experiments with similar results. **(B)**. HUVEC subjected to co-immunoprecipitation with the antibody against immunoglobulin G (**IgG**) or STAT3 (**STAT3**) followed by Western blotting using indicated antibodies. Results shown were from a representative experiment of three independent experiments with similar results. **(C)**. Quantification of results in (**Fig 5D**). The quantified results represent the mean ± SD. Three independent experiments were performed and similar results were obtained, each experiment containing seven technical replicates.
(TIF)

S4 Fig Screening and identification of lentivirus-mediated short hairpin RNA targeting STAT3. Western blotting was performed in HUVEC transduced with lentivirus-mediated No.1 (**sh1STAT3**), No. 2 (**sh2STAT3**), No. 3 (**sh3STAT3**), and a mixture of No. 1, 2, and 3 together (**shSTAT3**) of short hairpin RNAs targeting STAT3 or the control (**mpCDH**) with the indicated antibodies. Results shown were from a representative experiment of three independent experiments with similar results. The values of density of protein bands after normalization to housekeeping were shown.
(TIF)

S5 Fig Deletion of miR-K6 from the KSHV genome does not affect the expression of other miRNAs. **(A)**. Total RNA was extracted from HUVEC infected with BAC16 KSHV wide type virus (KSHV_WT) or BAC16 KSHV miR-K6 deletion mutant virus (miR-K6_Mut), and levels of KSHV miRNAs miR-K3, -K4-3p, -K6-3p, and -K6-5p were measured using qPCR. The quantified results represent the mean ± SD. Three independent experiments were performed and similar results were obtained, each experiment containing four technical replicates. Undet., undetermined. *n*.*s*., not significant. **(B)**. The mRNA expression of LANA in HUVEC treated as in (**A**). The quantified results represent the mean ± SD. Three independent experiments were performed and similar results were obtained, each experiment containing four technical replicates. *n*.*s*., not significant.
(TIF)

S6 Fig Knockdown of miR-K6-3p from KSHV genome attenuates KSHV induction of endothelial cell migration and angiogenesis. **(A)**. Western blotting analysis of expression of SH3BGR, phosphorylated STAT3 and STAT3 in HUVEC treated with PBS (**PBS**), infected with BAC16 KSHV wide type virus (**KSHV_WT**) and further transduced with miR-K6-3p inhibitor (**miR-K6-3p inhibitor**). Results shown were from a representative experiment of three independent experiments with similar results. The values of density of protein bands after normalization to housekeeping were shown. **(B)**. Transwell migration assay for HUVEC treated as in (**A**). The quantified results represent the mean ± SD. Three independent experiments were performed and similar results were obtained, each experiment containing four technical replicates. * *P* < 0.05, ** *P* < 0.01, and *** *P* < 0.001 for Student’s *t*-test. **(C)**. Microtubule formation assay for HUVEC treated as in (**A**). The quantified results represent the mean ± SD. Three independent experiments were performed and similar results were obtained, each experiment containing five technical replicates. ** *P* < 0.01, and *** *P* < 0.001 for Student’s t-test.
(TIF)

S7 Fig Screening and identification of lentivirus-mediated short hairpin RNA targeting SH3BGR. Western blotting was performed in HUVEC transduced with lentivirus-mediated No.1 (**sh1SH3BGR**), No. 2 (**sh2SH3BGR**), No. 3 (**sh3SH3BGR**), No. 4 (**sh4SH3BGR**) and a mixture of No. 2, 3, and 4 together (**shSH3BGR**) of short hairpin RNAs targeting SH3BGR or the control (**mpCDH**) with the indicated antibodies. Results shown were from a representative experiment of three independent experiments with similar results. The values of density of protein bands after normalization to housekeeping were shown.
(TIF)

S8 Fig MiR-K6-3p is necessary for KSHV-induced endothelial cell migration and angiogenesis. **(A)**. Western blotting analysis of expression of SH3BGR, phosphorylated STAT3 and STAT3 in HUVEC infected with BAC16 KSHV wide type virus (**KSHV_WT**) or BAC16 KSHV miR-K6 deletion mutant virus (**miR-K6_Mut**) and further transduced with 1 MOI lentivirus empty vector (**mpCDH**) or lentivirus-miR-K6-3p (**miR-K6-3p**). Results shown were from a representative experiment of three independent experiments with similar results. The values of density of protein bands after normalization to housekeeping were shown. **(B)**. Transwell migration assay for HUVEC treated as in (**A**). The quantified results represent the mean ± SD. Three independent experiments were performed and similar results were obtained, each experiment containing five technical replicates. * *P* < 0.05, ** *P* < 0.01, and *** *P* < 0.001 for Student’s *t*-test. **(C)**. Microtubule formation assay for HUVEC treated as in (**A**). The quantified results represent the mean ± SD. Three independent experiments were performed and similar results were obtained, each experiment containing four technical replicates. * *P* < 0.05, ** *P* < 0.01, and *** *P* < 0.001 for Student’s *t*-test.
(TIF)
