# Supplementary material for: The SH3BGR/STAT3 Pathway Regulates Cell Migration and Angiogenesis Induced by a Gammaherpesvirus MicroRNA
Source: PLoS Pathog. 2016 Apr 29;12(4):e1005605. doi: 10.1371/journal.ppat.1005605 (PMC4851422; doi:10.1371/journal.ppat.1005605)
Supplement: S1 Table — (DOCX) [file ppat.1005605.s001.docx]

**S1 Table**. A list of accession numbers/ID numbers for genes mentioned in the text.

| Gene name | GENE ID |
| --- | --- |
| SH3BGR | 6450 |
| STAT3 | 6774 |
| VEGFA | 7422 |
| VEGFR2 | 3791 |
| MMP1 | 4312 |
| MMP2 | 4313 |
| MMP3 | 4314 |
| MMP7 | 4316 |
| MMP9 | 4318 |
| MMP13 | 4322 |
| IL6 | 3569 |
| IL8 | 3576 |
| BACH1 | 571 |
| MAF | 4094 |
| P21 | 1026 |
| NFIB | 4781 |
| MYB | 4602 |
| C/EBPα | 140815 |
| Ets-1 | 2113 |
| GRK2 | 156 |
| SMA | 59 |
| KSHV LANA | 4961527 |
| KSHV K15 | 4961473 |
| KSHV vIL6 | 4961449 |
| KSHV RTA | 4961526 |
