# Supplementary material for: The SH3BGR/STAT3 Pathway Regulates Cell Migration and Angiogenesis Induced by a Gammaherpesvirus MicroRNA
Source: PLoS Pathog. 2016 Apr 29;12(4):e1005605. doi: 10.1371/journal.ppat.1005605 (PMC4851422; doi:10.1371/journal.ppat.1005605)
Supplement: S2 Table — (DOCX) [file ppat.1005605.s002.docx]

**S2 Table**. A list of accession numbers/ID numbers for miRNAs mentioned in the text.

| microRNA | Accession number |
| --- | --- |
| KSHV-miR-K6-3p | [MIMAT0002189](http://mirbase.org/cgi-bin/mature.pl?mature_acc=MIMAT0002189) |
| KSHV-miR-K3 | MIMAT0002193 |
| KSHV-miR-K6-5p | [MIMAT0002188](http://mirbase.org/cgi-bin/mature.pl?mature_acc=MIMAT0002189) |
| KSHV-miR-K1 | MIMAT0002182 |
| KSHV-miR-K4-3p | MIMAT0002192 |
| KSHV-miR-K11 | MIMAT0002181 |
| hsa-miR-155 | MIMAT0000646 |
