# Supplementary figures and images for: The SH3BGR/STAT3 Pathway Regulates Cell Migration and Angiogenesis Induced by a Gammaherpesvirus MicroRNA
Source: PLoS Pathog. 2016 Apr 29;12(4):e1005605. doi: 10.1371/journal.ppat.1005605 (PMC4851422; doi:10.1371/journal.ppat.1005605)

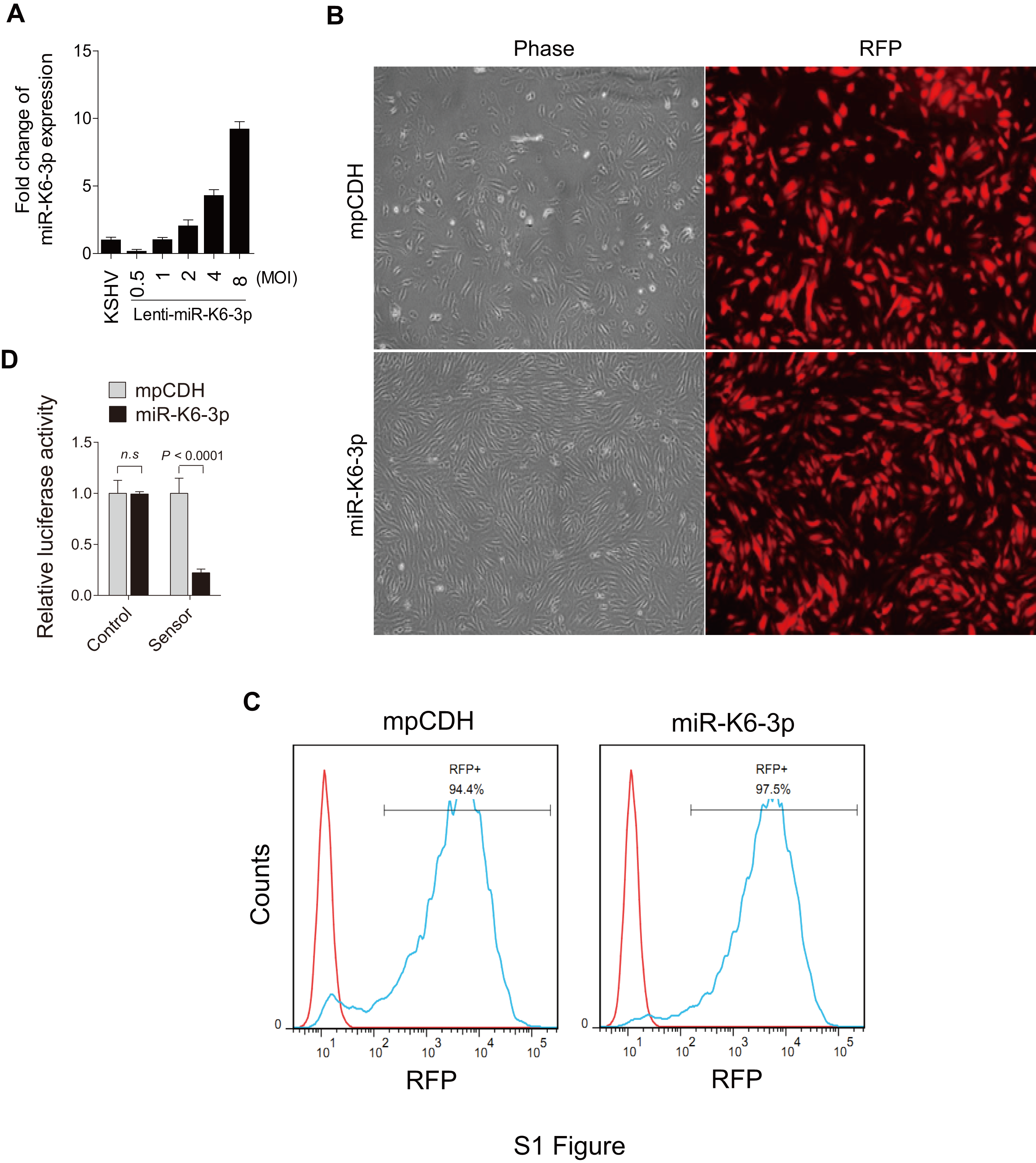

Supplement: S1 Fig — (A). KSHV miR-K6-3p expression in HUVEC infected with KSHV BAC16 virus induced from iSLK-BAC16 cells or transduced by the different MOI of lentiviral miR-K6-3p were determined by RT-qPCR. The miR-K6-3p level in KSHV group was set as ‘‘1” for comparison. The quantified results represent the mean ± SD. Three independent experiments were performed and similar results were obtained, each experiment containing four technical replicates. (B). HUVEC were transduced with 1 MOI lentivirus empty vector (mpCDH; top) and lentivirus-miR-K6-3p (miR-K6-3p; bottom), and representative images were taken under the light microscope (Phase; left) and fluorescent microscope (RFP; right) (Original magnification, ×100). (C). HUVEC were transduced with 1 MOI of lentivirus empty vector (mpCDH; left) and lentivirus-miR-K6-3p (miR-K6-3p; right) were analyzed for RFP expression by flow cytometry to determine transduction efficiency. (D). Luciferase activity was detected in 1 MOI of lentivirus empty vector (mpCDH) or lentivirus-miR-K6-3p (miR-K6-3p) transduced HUVEC transfected by the pGL3-Control (Control) or the pGL3-miR-K6-3p sensor reporter (Sensor). n.s., not significant. (TIF) [file ppat.1005605.s003.tif]

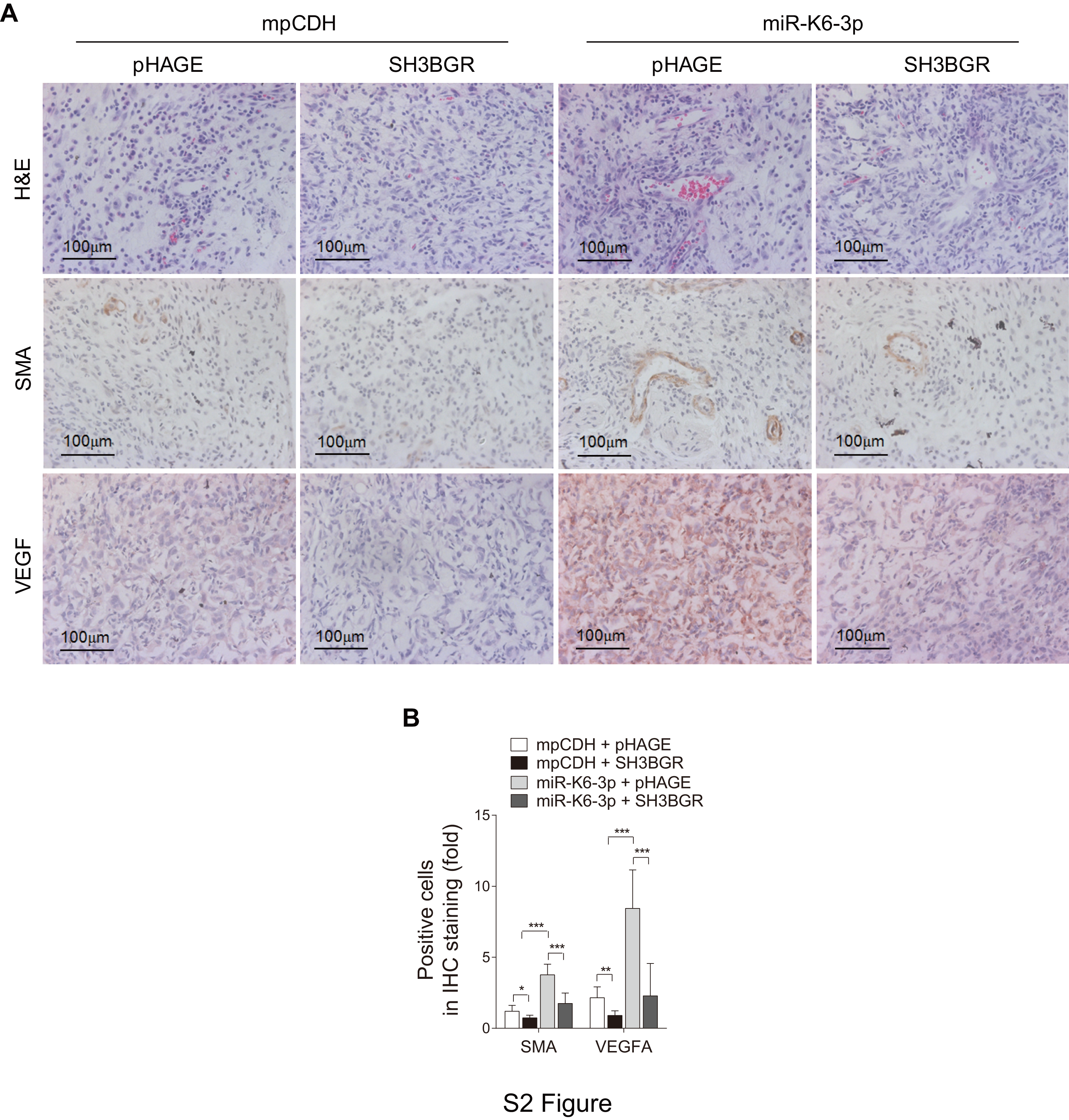

Supplement: S2 Fig — (A). The Matrigel plugs treated as in (Fig 4G) were fixed, sectioned, and stained with hematoxylin and eosin (top; original magnification, x400), SMA (middle; original magnification, x400) and VEGFA (bottom; original magnification, x400). (B). Quantification of results in (A). ** P < 0.01 and *** P < 0.001 for Student’s t-test. (TIF) [file ppat.1005605.s004.tif]

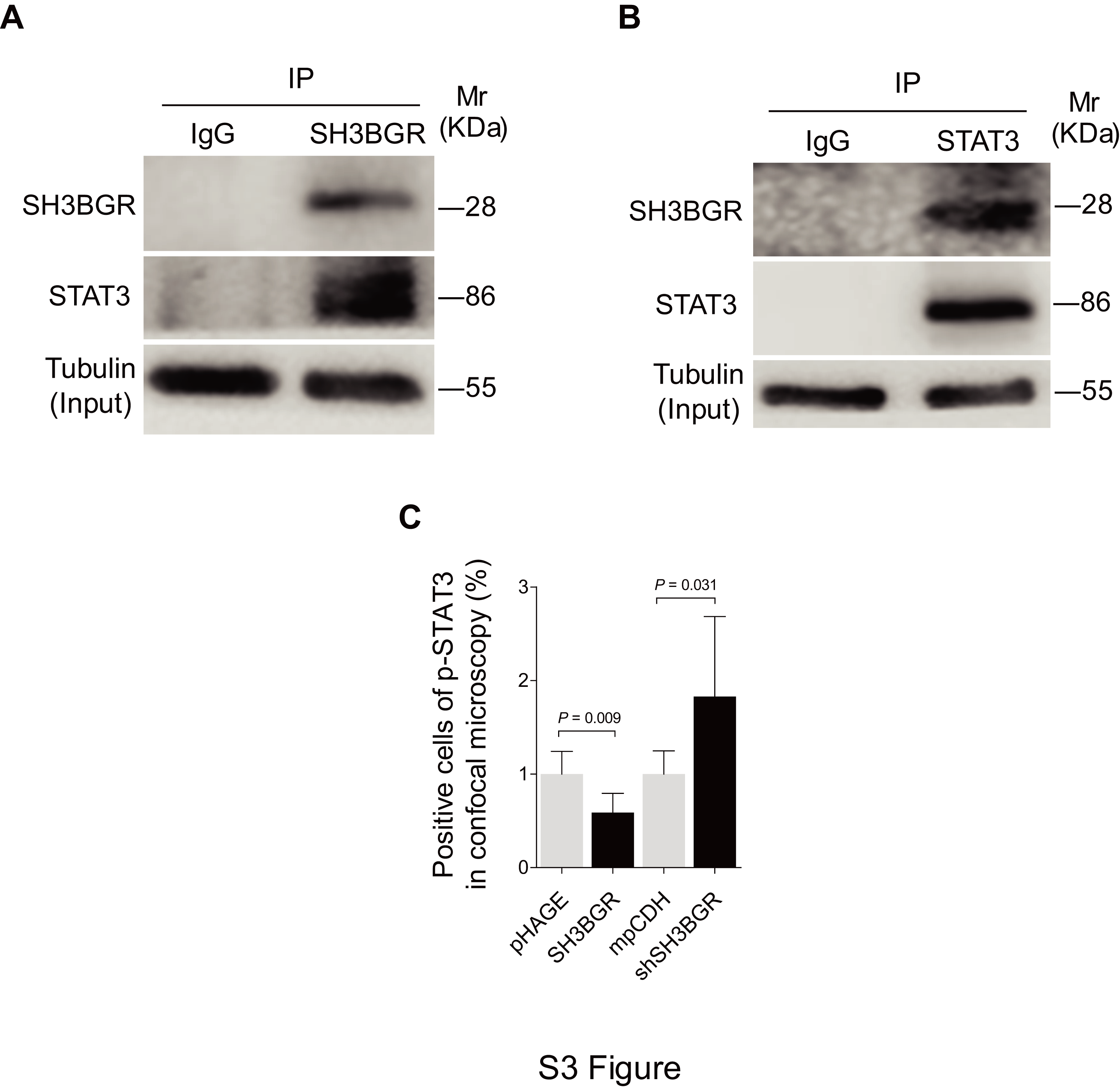

Supplement: S3 Fig — (A). HUVEC subjected to co-immunoprecipitation with the antibody against immunoglobulin G (IgG) or SH3BGR (SH3BGR) followed by Western blotting using indicated antibodies. Results shown were from a representative experiment of three independent experiments with similar results. (B). HUVEC subjected to co-immunoprecipitation with the antibody against immunoglobulin G (IgG) or STAT3 (STAT3) followed by Western blotting using indicated antibodies. Results shown were from a representative experiment of three independent experiments with similar results. (C). Quantification of results in (Fig 5D). The quantified results represent the mean ± SD. Three independent experiments were performed and similar results were obtained, each experiment containing seven technical replicates. (TIF) [file ppat.1005605.s005.tif]

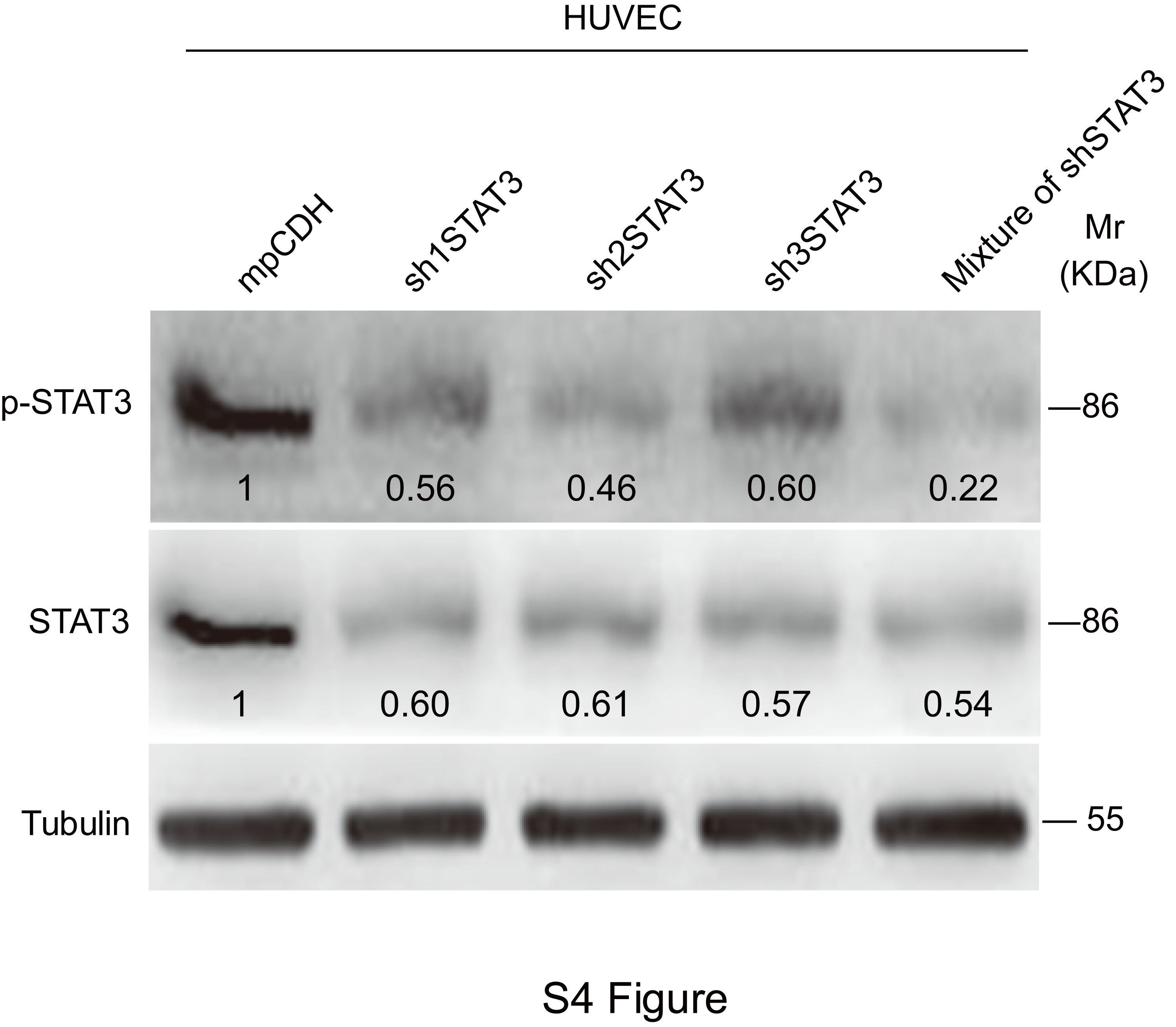

Supplement: S4 Fig — Western blotting was performed in HUVEC transduced with lentivirus-mediated No.1 (sh1STAT3), No. 2 (sh2STAT3), No. 3 (sh3STAT3), and a mixture of No. 1, 2, and 3 together (shSTAT3) of short hairpin RNAs targeting STAT3 or the control (mpCDH) with the indicated antibodies. Results shown were from a representative experiment of three independent experiments with similar results. The values of density of protein bands after normalization to housekeeping were shown. (TIF) [file ppat.1005605.s006.tif]

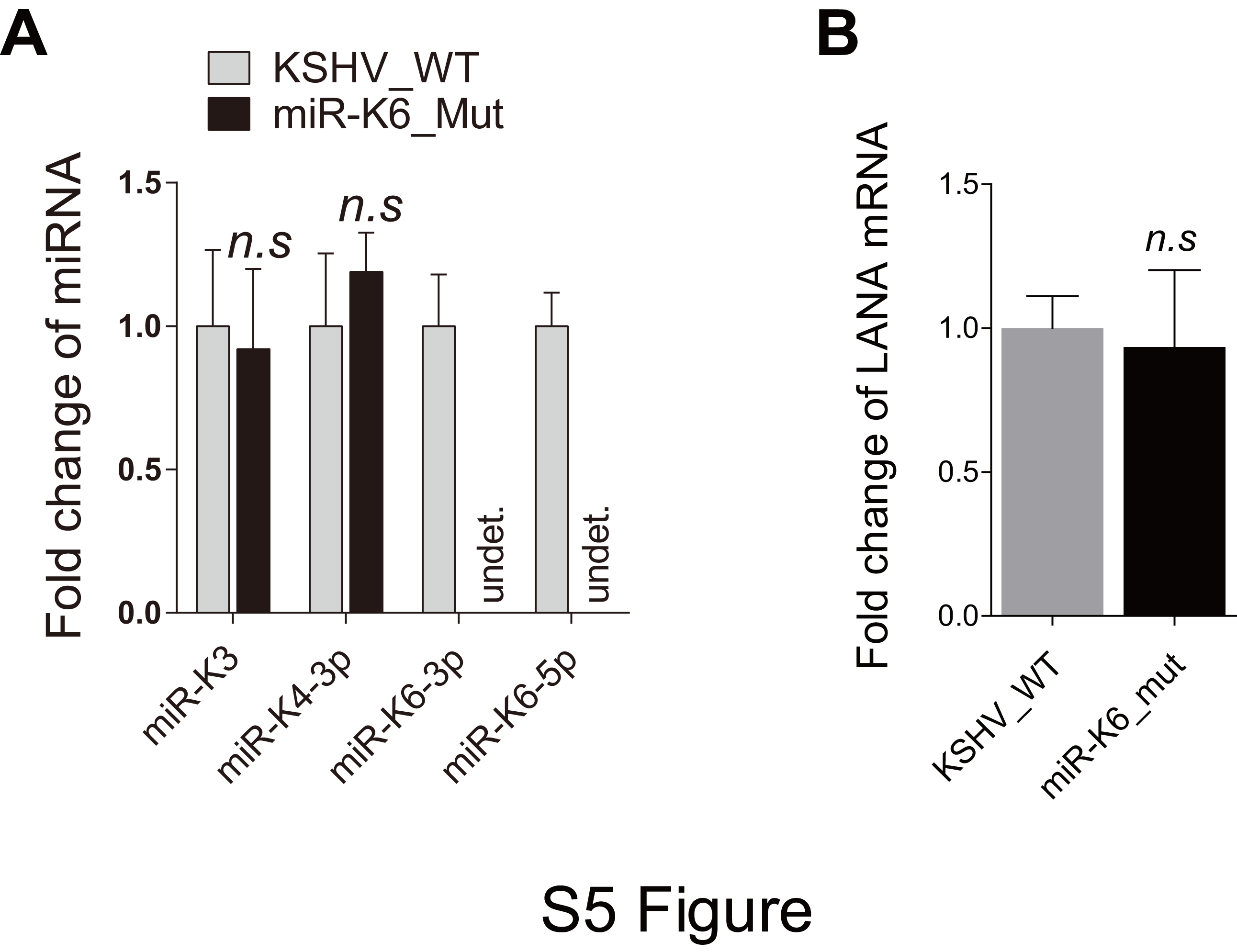

Supplement: S5 Fig — (A). Total RNA was extracted from HUVEC infected with BAC16 KSHV wide type virus (KSHV_WT) or BAC16 KSHV miR-K6 deletion mutant virus (miR-K6_Mut), and levels of KSHV miRNAs miR-K3, -K4-3p, -K6-3p, and -K6-5p were measured using qPCR. The quantified results represent the mean ± SD. Three independent experiments were performed and similar results were obtained, each experiment containing four technical replicates. Undet., undetermined. n.s., not significant. (B). The mRNA expression of LANA in HUVEC treated as in (A). The quantified results represent the mean ± SD. Three independent experiments were performed and similar results were obtained, each experiment containing four technical replicates. n.s., not significant. (TIF) [file ppat.1005605.s007.tif]

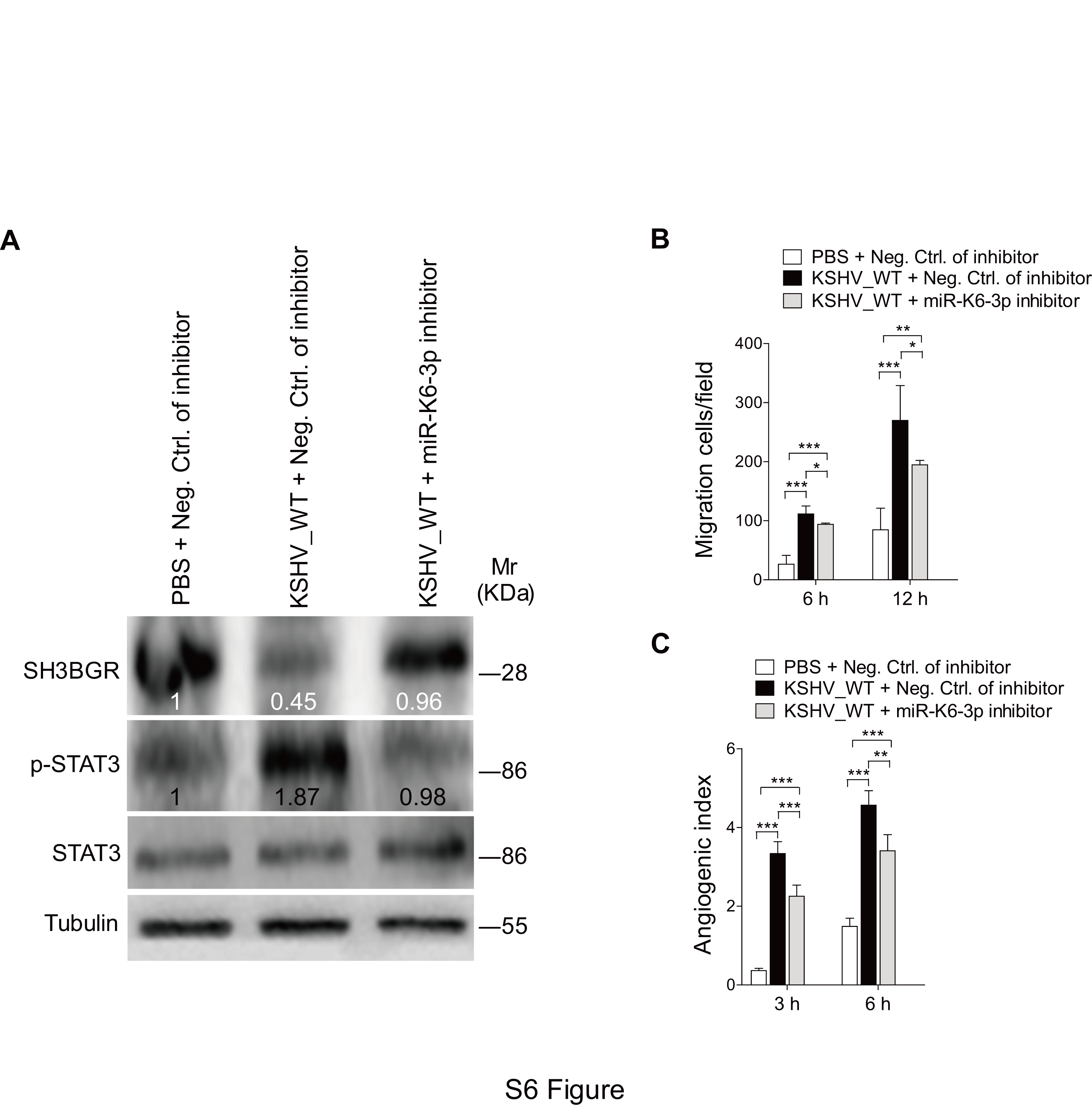

Supplement: S6 Fig — (A). Western blotting analysis of expression of SH3BGR, phosphorylated STAT3 and STAT3 in HUVEC treated with PBS (PBS), infected with BAC16 KSHV wide type virus (KSHV_WT) and further transduced with miR-K6-3p inhibitor (miR-K6-3p inhibitor). Results shown were from a representative experiment of three independent experiments with similar results. The values of density of protein bands after normalization to housekeeping were shown. (B). Transwell migration assay for HUVEC treated as in (A). The quantified results represent the mean ± SD. Three independent experiments were performed and similar results were obtained, each experiment containing four technical replicates. * P < 0.05, ** P < 0.01, and *** P < 0.001 for Student’s t-test. (C). Microtubule formation assay for HUVEC treated as in (A). The quantified results represent the mean ± SD. Three independent experiments were performed and similar results were obtained, each experiment containing five technical replicates. ** P < 0.01, and *** P < 0.001 for Student’s t-test. (TIF) [file ppat.1005605.s008.tif]

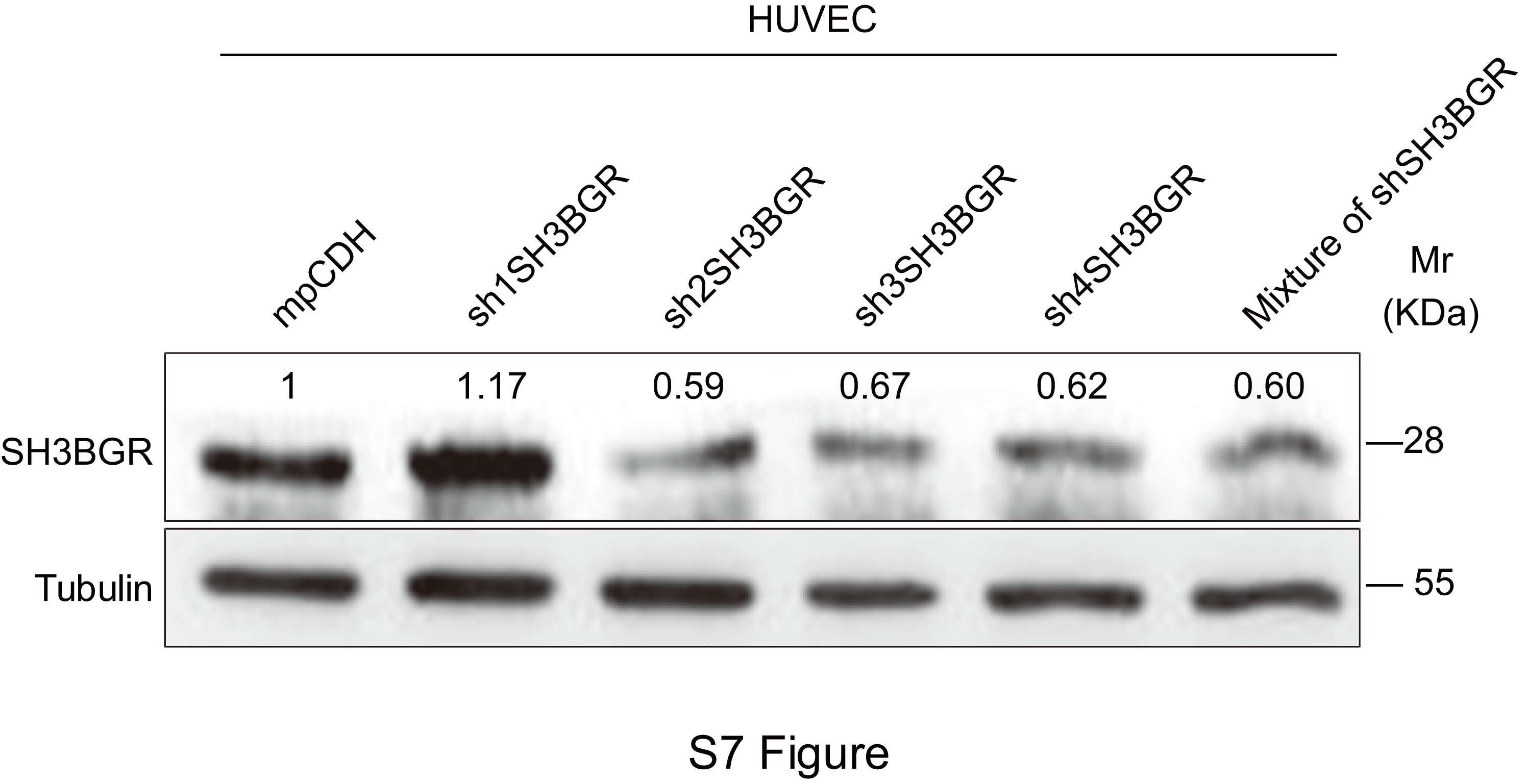

Supplement: S7 Fig — Western blotting was performed in HUVEC transduced with lentivirus-mediated No.1 (sh1SH3BGR), No. 2 (sh2SH3BGR), No. 3 (sh3SH3BGR), No. 4 (sh4SH3BGR) and a mixture of No. 2, 3, and 4 together (shSH3BGR) of short hairpin RNAs targeting SH3BGR or the control (mpCDH) with the indicated antibodies. Results shown were from a representative experiment of three independent experiments with similar results. The values of density of protein bands after normalization to housekeeping were shown. (TIF) [file ppat.1005605.s009.tif]

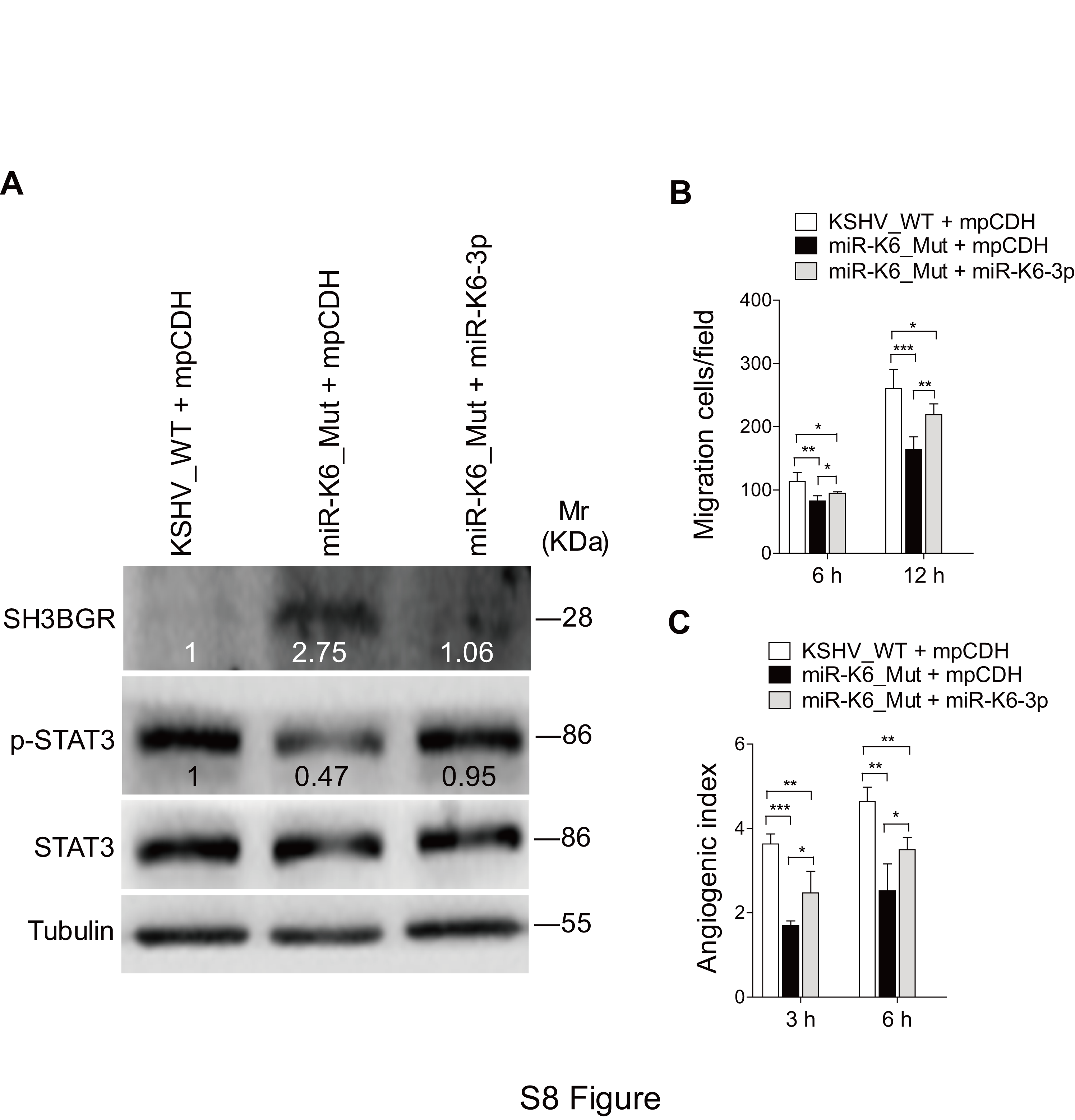

Supplement: S8 Fig — (A). Western blotting analysis of expression of SH3BGR, phosphorylated STAT3 and STAT3 in HUVEC infected with BAC16 KSHV wide type virus (KSHV_WT) or BAC16 KSHV miR-K6 deletion mutant virus (miR-K6_Mut) and further transduced with 1 MOI lentivirus empty vector (mpCDH) or lentivirus-miR-K6-3p (miR-K6-3p). Results shown were from a representative experiment of three independent experiments with similar results. The values of density of protein bands after normalization to housekeeping were shown. (B). Transwell migration assay for HUVEC treated as in (A). The quantified results represent the mean ± SD. Three independent experiments were performed and similar results were obtained, each experiment containing five technical replicates. * P < 0.05, ** P < 0.01, and *** P < 0.001 for Student’s t-test. (C). Microtubule formation assay for HUVEC treated as in (A). The quantified results represent the mean ± SD. Three independent experiments were performed and similar results were obtained, each experiment containing four technical replicates. * P < 0.05, ** P < 0.01, and *** P < 0.001 for Student’s t-test. (TIF) [file ppat.1005605.s010.tif]
